# Aligned Conductive Magnetic Nanofibers with Directional Magnetic Field Stimulation Promotes Peripheral Nerve Regeneration

**DOI:** 10.1002/advs.202501665

**Published:** 2025-07-06

**Authors:** Zheyuan Fan, Wei Yu, Xinggui Wen, Xiangdong Ding, Xiang Li

**Affiliations:** ^1^ Department of Wound Repair Plastic and Reconstructive Microsurgery, China‐Japan Union Hospital Jilin University Changchun 130033 P. R. China; ^2^ MacDiarmid Institute, College of Chemistry Jilin University Changchun 130012 P. R. China

**Keywords:** aligned fibrin nanofibers, conducting material, magnetic field stimulation, magnetic nanoparticles, peripheral nerve regeneration

## Abstract

Peripheral nerve injury is one of the most common disorders of the nervous system. Alternatives to autologous nerve transplantation have attracted significant interest among researchers. In this study, magnetic nanoparticles are integrated with oriented polycaprolactone (PCL) fibers, followed by the addition of a polypyrrole (Ppy) coating. Ppy‐PCL/Fe_3_O_4_, when combined with a static magnetic field, activates the superparamagnetic properties of the nanoparticles while ensuring conductivity, creating an environment conducive to nerve regeneration. The optimal intensity of the external magnetic field stimulation is assessed in vitro, and its effects on calcium influx and differentiation in rat RSC96 and PC12 cells, respectively, are examined. The superior efficacy of the integrated system in nerve regeneration is confirmed by histological and functional analyses in vivo. Exploration of the underlying molecular pathways using transcriptome sequencing shows that the regenerative system promotes the release of brain‐derived neurotrophic factor and reduces the production of reactive oxygen species. This comprehensive approach not only demonstrates the efficacy of the system in promoting peripheral nerve regeneration but also lays the groundwork for elucidating the underlying mechanistic pathways involved.

## Graphical abstract

Peripheral nerve injury necessitates alternatives to autografts. This study combines magnetic nanoparticles, oriented PCL fibers, and Ppy to create a conductive, magnetically active scaffold. In vitro and in vivo experiments demonstrated enhanced downstream pathways of calcium signaling, as revealed by transcriptome analysis. A promising strategy for nerve repair.

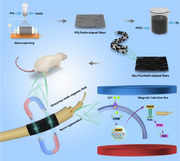

## 1 Introduction

Peripheral nerve injury (PNI) is the most common traumatic injury in the nervous system.^[^ ^1^ ^]^ The repair of long‐segment neurological defects caused by PNI is particularly challenging^[^ ^2^ ^]^ and is usually performed using autologous nerve grafting, currently considered the gold standard in such cases.^[^ ^3^ ^]^ However, this approach has several limitations, including shortages of donor nerves, size mismatches, and increased morbidity at the donor sites, and researchers are continuously seeking viable alternatives.^[^ ^4^ ^]^ Over the past few decades, various types of nerve conduits with suitable mechanical properties and biocompatibility have been designed for targeted regeneration of the nerve fibers.^[^ ^5^ ^]^

The potential use of magnetism as a directional cue for cellular growth has attracted attention within the scientific community. A pioneering study by Murayama et al. in 1965 observed that sickled red blood cells orient perpendicular to an applied magnetic field.^[^ ^6^ ^]^ This seminal discovery led to subsequent investigations into the interactions between magnetic fields and various types of cells, including red blood cells, fibroblasts, osteoblasts, and human glioblastoma A172 cells, as well as collagen fibers.^[^ ^7^ ^]^ Notably, research conducted by Xx et al. revealed that rat pheochromocytoma PC12 cells, when stimulated by a static magnetic field and nerve growth factor (NGF), exhibited chemotactic axonal extension perpendicular to the magnetic vector. Given neuromorphic characteristics of PC12 cells, these findings provide a theoretical basis for the utilization of magnetic fields in creating a microenvironment that favors peripheral nerve regeneration.

The use of magnetic nanoparticles as scaffolds for diverse biomaterials has attracted significant interest.^[^ ^8^ ^]^ The integration of magnetic nanoparticles with exosomes or alternative metallic nanoparticles, as well as their application as precise navigational units, represents a notable area of research. Many studies have evaluated the use of magnetic nanoparticles, especially in conjunction with an external magnetic field, as “carriers” or “delivery agents” for targeted therapeutic applications and the controlled delivery of bioactive agents. For instance, Liu et al. developed a fluorescent‐magnetic bifunctional superparamagnetic iron oxide nanoparticle (SPION) framework that was found to be effective in targeting the sciatic nerve. Their findings suggested that SPION‐driven magnetic guidance contributed significantly to the induction and maintenance of Schwann cell phenotypes that supported nerve repair, thus facilitating the structural and functional restoration of the sciatic nerve following compressive trauma. Similarly, Clara et al. used a similar approach, incorporating adipose‐derived mesenchymal stem cells with magnetic targeting nanoparticles to promote cell homing to damaged nerves, and subsequent enhancement of remyelination and repair of the nerves. Beyond their use in cutting‐edge biomaterials, magnetic nanoparticles also have significant promise as primary therapeutic entities. The controlled hyperthermia resulting from magnetization due to alternating magnetic fields has been applied in preliminary investigations into targeting tumor ablation, illustrating the therapeutic potential of the approach. The application of magnetic biosensors has attracted interest in the field of biosensing modalities. Research into nerve regeneration through the use of superparamagnetic phenomena induced by the application of external magnetic fields to magnetic nanoparticles has been conducted largely in vitro. Qin et al. synthesized nerve growth factor‐functionalized superparamagnetic iron oxide gold (NGF‐SPIO‐Au) nanoparticles, which, in synergy with an external magnetic field, promoted neuronal differentiation by inducing the influx of calcium ions and subsequent membrane depolarization. A further study embedded magnetic nanoparticles within fibrous structures to utilize their superparamagnetic capabilities.^[^ ^9^ ^]^ A variety of materials have been employed in the fabrication of magnetic nanofibers, including poly(ε‐caprolactone) (PCL), hydroxyapatite (HA), magnetic poly (L‐lactic acid) (PLLA), and poly (glycolide‐co‐lactide) (PGLA). Gilbert et al. investigated the ability of magnetic nanoparticle scaffolds used in conjunction with magnetic fields to enhance neurite outgrowth. This approach relied on both guidance of contact between aligned fibers and the stimulation provided by the magnetic nanoparticles within the magnetic field, demonstrating the synergistic effects of structural support and magnetic stimulation in promoting neural regeneration, offering insights into the utility of magnetic nanoparticle scaffolds in neural tissue engineering applications.^[^ ^9^ ^]^

The facilitation of neural regeneration by aligned fibers has been confirmed by numerous studies. Bellamkonda et al. were the first to demonstrate the beneficial effects of the submicron alignment of polymer fibers on peripheral nerve regeneration.^[^ ^10^ ^]^ Subsequently, aligned fibers have emerged as the preferred architecture for electrospun nerve conduits, and a growing body of research has focused on the augmentation of aligned fibers with a variety of functional cells and growth factors. These studies have shown significant enhancement in axonal regeneration, with increased numbers of both regenerating axons and dorsal root ganglion neurons, together with improved functional recovery of target organs facilitated by electrical stimulation. A study by Tang et al. also observed that electrical stimulation markedly accelerated the clearance of damaged axons and myelin and promoted Schwann cell dedifferentiation, thereby hastening the initial stages of axonal and vascular regeneration.^[^ ^11^ ^]^ A clinical investigation showed that treatment with 20 Hz pulsed electrical stimulation (ES) significantly enhanced recovery of function in transected sensory nerves in the hand, with notable improvements observed six months post‐operation. The integration of conductive guidance fibers has been found to be markedly effective in promoting axonal extension,^[^ ^12^ ^]^ highlighting the synergistic potential of combining structural and electrical cues in nerve repair strategies.

In this study, magnetic nanoparticles were incorporated with aligned PCL fibers and further enhanced with a polypyrrole (Ppy) coating to confer conductivity to the nanofiber membrane. This innovative biomaterial when used with a static magnetic field activated the superparamagnetic features of the embedded magnetic nanoparticles. This dual biofunctional approach, including both biomagnetic and bioelectric elements, provides an environment conducive to the regeneration of nearby nerve injuries. The optimal intensity of the external magnetic field stimulation was determined in vitro, together with an investigation of its effects on the influx of intracellular calcium in rat Schwann RSC96 cells and on differentiation in PC12 cells. In vivo experiments using autologous nerve grafts, PCL‐based constructs, and controls not exposed to magnetic fields showed superior structural and functional recovery in nerves treated with this system. Transcriptome sequencing was also used to examine the mechanisms underlying the regenerative process. This comprehensive approach not only demonstrated the efficacy of the system in promoting peripheral nerve regeneration but also laid the groundwork for elucidating the molecular pathways involved.

## 2 Results and Discussion

### 2.1 Characterization of Ppy‐PCL/Fe_3_O_4_

**Figure 1:**
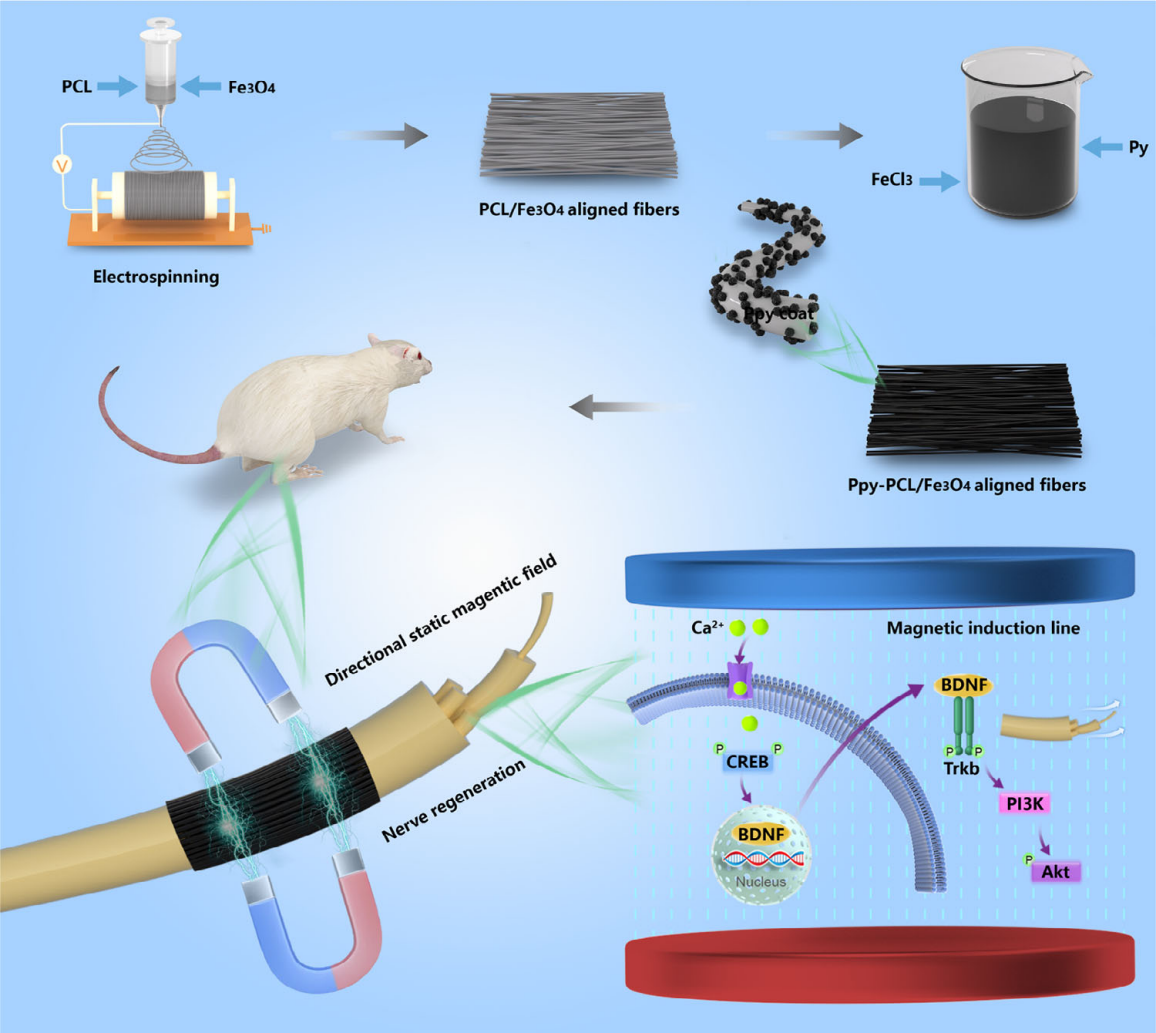
A schematic representation of a nerve conduit integrated with aligned magnetic nanoparticle fibers under an axial magnetic field. This strategy stimulates the influx of calcium ions in Schwann cells, promotes CREB phosphorylation, and enhances BDNF secretion in Schwann cells, thereby inducing Trkb and Akt phosphorylation, and enhancing directed axonal extension.

**Figure 2:**
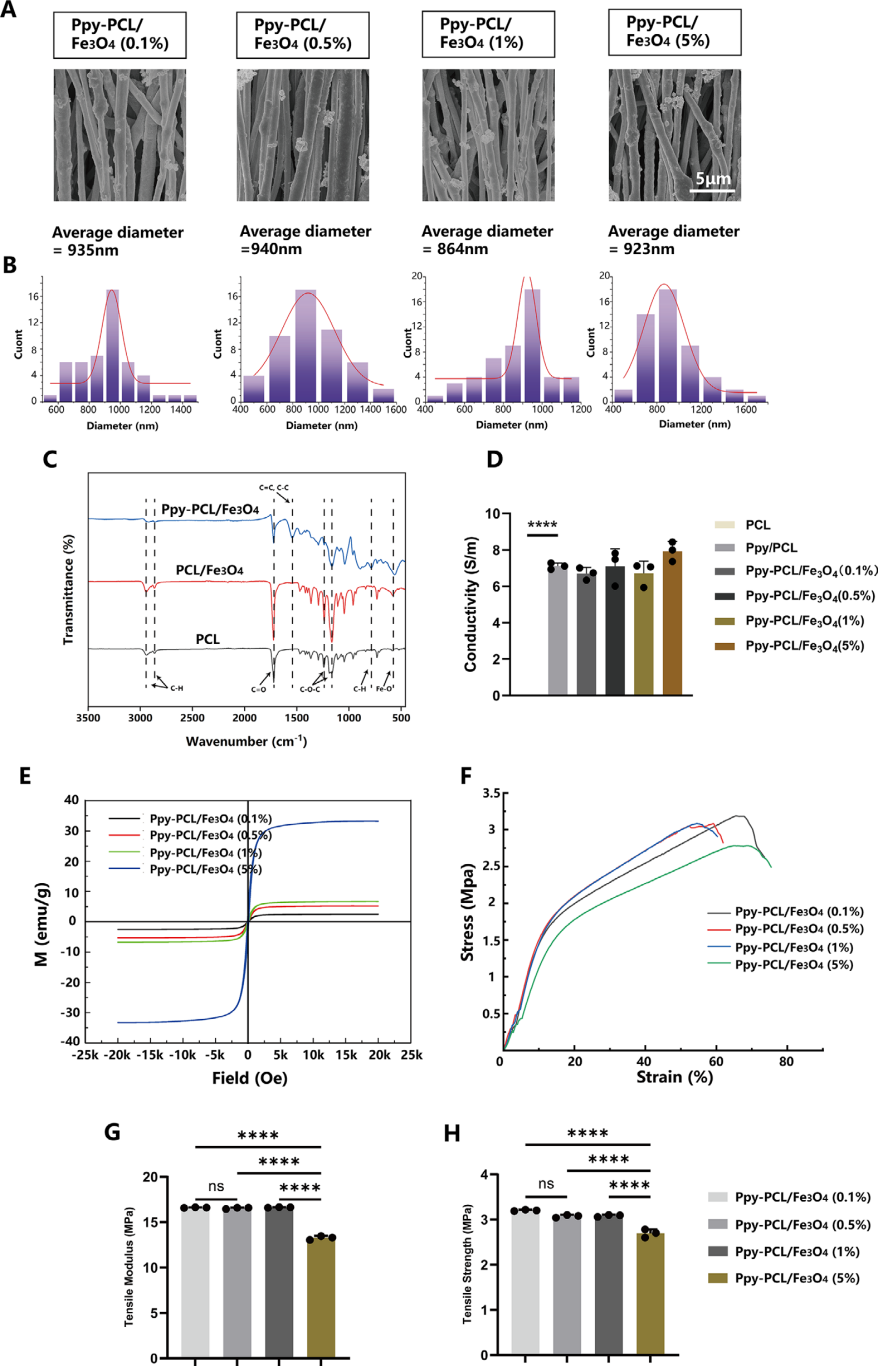
A) Scanning electron microscopy images of the fiber membranes. B) Distribution of fiber membrane diameters. C) FT‐IR spectra of PCL, PCL/ Fe_3_O_4_, and Ppy‐PCL/ Fe_3_O_4_ nanofibers. D) Conductivity of PCL, Ppy/PCL, Ppy‐PCL/ Fe_3_O_4_ (0.1%, 0.5%, 1%, and 5%), n=3. E) Hysteresis loop analysis of Ppy‐PCL/ Fe_3_O_4_ (0.1%, 0.5%, 1%, and 5%). F) Stress‐strain curves of Ppy‐PCL/ Fe_3_O_4_ (0.1%, 0.5%, 1%, and 5%). G) Tensile modulus of Ppy‐PCL/ Fe_3_O_4_ (0.1%, 0.5%, 1%, and 5%). H) Tensile strength of Ppy‐PCL/ Fe_3_O_4_ (0.1%, 0.5%, 1%, and 5%), n=3. Data are expressed as mean ± SD. Statistical analysis was performed using one‐way ANOVA (D, G, H). ^*^ *p* < 0.05; ^**^ *p *< 0.01; ^***^ *p *< 0.001; ^****^ *p *< 0.0001; ns, no significance.

The directed neural induction system using a static magnetic field is shown in **Figure** **1**. In this study, a novel material (Ppy‐PCL/Fe_3_O_4_) was prepared to promote growth cone extension and guidance by mixing magnetic nanoparticles with PCL, based on classic strategies for promoting nerve regeneration, namely, the alignment of fibers and conductivity. Color differences were visible before the addition of the Ppy coating, with the color deepening gradually as the content of magnetic nanoparticles increased (Figure S1, Supporting Information). In situ polymerization was used to coat the surface of the PCL/Fe_3_O_4_ with a layer of Ppy to enhance the conductivity of the insulating PCL material. No significant differences were observed between the different groups after Ppy polymerization, with all showing a uniform black color (Figure S2, Supporting Information). **Figure** **2**C shows the infrared spectra of PCL, PCL/Fe_3_O_4_, and Ppy‐PCL/Fe_3_O_4_. The peaks at 2943 and 2864 cm^−1^ correspond to the asymmetric and symmetric stretching, respectively, of C─H bonds in the PCL. The peak at 1721 cm^−1^ corresponds to C═O stretching of the ester carbonyl group in the PCL,^[^ ^13^ ^]^ while the peaks at 1239 and 1160 cm^−1^ correspond to the asymmetric and symmetric stretching, respectively, of C‐O‐C in PCL.^[^ ^13^ ^]^ The peak at 578 cm^−1^ was attributed to the Fe─O band of the tetrahedral sites, confirming the spinel structure of Fe_3_O_4_.^[^ ^14^ ^]^ The characteristic peak at 1543 cm^−1^ was the result of stretching vibrations of the pyrrole ring, while the characteristic peak at 785 cm^−1^ was attributed to the stretching vibrations of C─H out‐of‐plane bending vibrations in the polypyrrole.^[^ ^15^, ^16^ ^]^ The X‐ray diffraction (XRD) spectrum of Ppy‐PCL/Fe_3_O_4_ is shown in Figure S3 (Supporting Information). The diffraction peaks at 2θ = 30.1, 35.5, 43.1, 57.1, and 62.7° correspond to the crystal planes (220), (311), (400), (511), and (440) of standard magnetite, respectively (Card No. 76‐0956).^[^ ^17^ ^]^ Scanning electron microscopy (SEM) was used to assess the morphology of fibers with different contents (0.1, 0.5, 1, 5wt%) of magnetic nanoparticles. Conductive nanofibers with different magnetic contents showed similar average diameters (0.1 wt.%: 935.06 ± 248.04 nm; 0.5 wt.%: 939.53±238.09 nm; 1 wt.%: 863.91±165.68 nm, 5 wt.%: 922.71 ± 182.89 nm) (Figure 2B). This result is consistent with those of previous reports.^[^ ^18^ ^]^ As seen with SEM, the Ppy appeared as dotted particles coated on the nanofibers, with any excess Ppy typically seen as nanostructures protruding from the fibers (Figure 2A). The TEM results show that the density of the magnetic nanoparticles increases with the increasing proportion of addition (Figure S4, Supporting Information). The hysteresis loops demonstrated superparamagnetic behavior in all the material groups, indicative of the magnetic response characteristics of the materials. The saturation magnetization strength corresponded to the concentration of magnetic nanoparticles in the different groups (Figure 2E). Ppy can form continuous films or coatings on the surfaces of various nanoparticles through in situ polymerization, thereby creating hybrid conductive fillers for use in polymer composite materials. A four‐point probe method was used to test the conductivity of four different materials. The results showed good conductivity in all groups, with no significant effects of the magnetic nanoparticle concentration on the conductivity of the material (Figure 2D). To determine whether this stable conductivity was maintained after activation of the superparamagnetism of the nanoparticles by an external magnetic field, the experiment was repeated under an external magnetic field of 500 Gs (Figure S4, Supporting Information). The results demonstrated that the conductivity remained stable with no significant difference compared to the lack of a magnetic field. The promotion of nerve regeneration by conductive materials has been confirmed by research,^[^ ^19^, ^20^ ^]^ showing that conductive nanofibers can induce the breakdown and clearance of damaged axons and myelin, as well as the dedifferentiation of Schwann cells, and facilitate the regeneration of axons and blood vessels.^[^ ^12^ ^]^ After in vivo implantation of the nano scaffolds, they are subjected to various mechanical actions, such as compression and traction from surrounding tissues, as well as activities such as cell migration and axon growth. It is therefore necessary for them to have sufficient strength and resilience to prevent excessive deformation that would adversely affect the long‐term structural stability of the scaffold and thus the ultimate repair outcome. The stress‐strain curves for each group are shown in Figure 2F. The elastic moduli of Ppy‐PCL/ Fe_3_O_4_ at concentrations of 0.1, 0.5, and 1 wt.% showed no statistical differences, while the elastic modulus at 5 wt.% was decreased. The results of the tensile strength assessment were consistent with those for the elastic modulus (Figure 2G,H). It can thus be concluded that incorporating magnetic nanoparticles at concentrations below 5 wt.% into PCL fibers results in optimal mechanical stress performance and provides sufficient protection for nerves within the conduit.

### 2.2 Ppy‐PCL/Fe_3_O_4_ In Vitro

**Figure 3:**
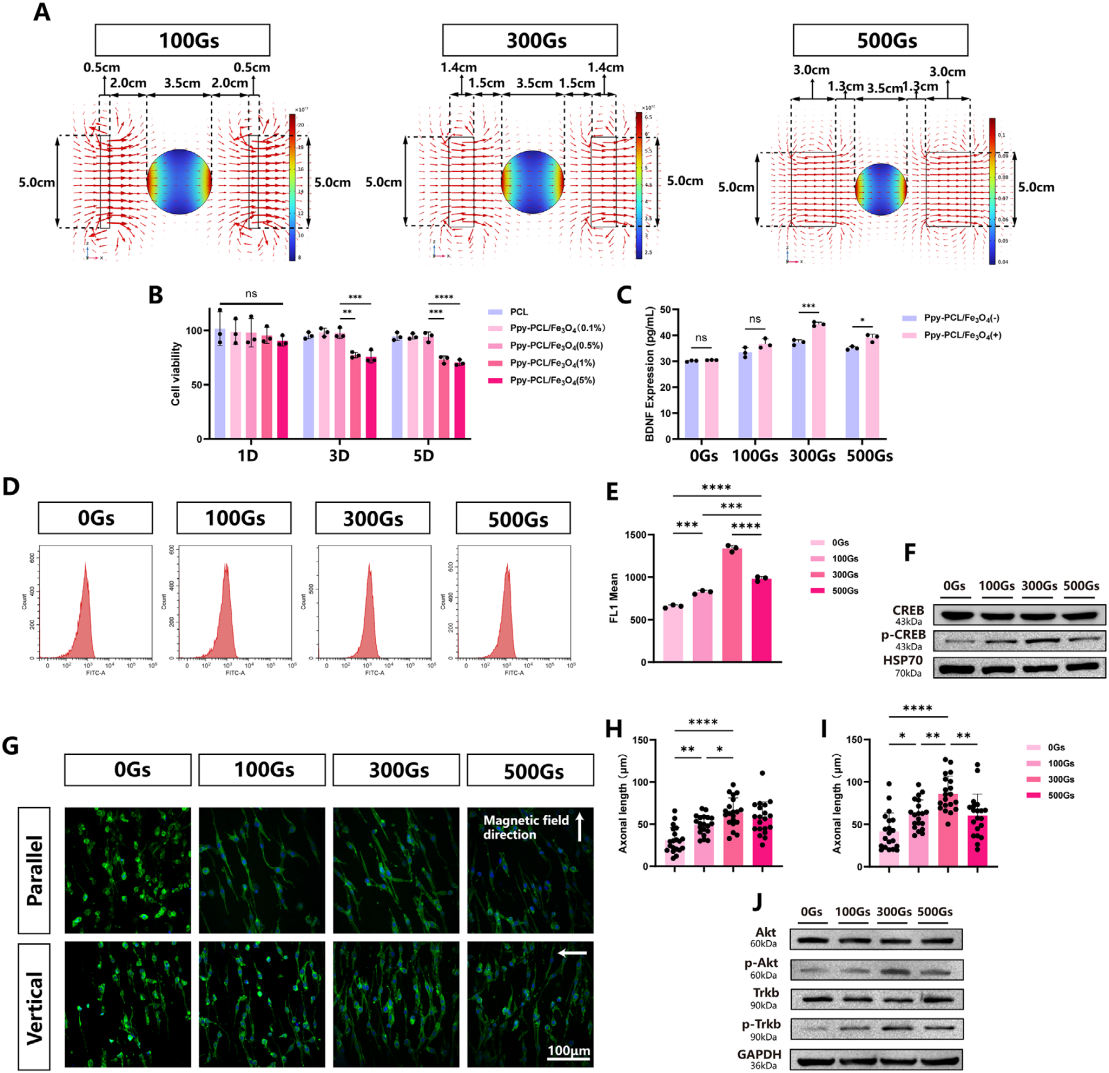
A) The colored diagram depicts the magnitude of the magnetic flux density (with the scale in milliteslas, mT), with the arrows showing the vector direction of the magnetic field. The circular area in the middle represents the position of the Petri dish. B) Cell viability of RSC96 cells treated with Ppy‐PCL/Fe_3_O_4_, n=3. C) Effects of Ppy‐PCL/Fe_3_O_4_ (0.5%) combined with magnetic fields of different strengths on BDNF expression in RSC96 cells, measured by ELISA, n=3. D) Flow cytometry analysis. E) Quantification and comparison of the mean fluorescence intensity of FL1 in RSC96 cells treated with Ppy‐PCL/Fe_3_O_4_ (0.5%) and different static magnetic fields, n=3. F) Expression levels of CREB and p‐CREB in RSC96 cells treated with Ppy‐PCL/Fe_3_O_4_ (0.5%) and different magnetic field strengths, n=3. G) Immunofluorescence images of PC12 cells on Ppy‐PCL/Fe_3_O_4_ (0.5%) with perpendicular or parallel magnetic fields. H) Comparison of axon lengths under parallel magnetic fields, n=20. I) Comparison of axon lengths under perpendicular magnetic fields, n=20. J) Expression of TrkB, p‐TrkB, Akt, and p‐Akt in PC12 cells treated with Ppy‐PCL/Fe_3_O_4_ (0.5%) and different magnetic field strengths, n=3. Statistical analysis was performed using one‐way ANOVA (B, E, H, I) and *two‐tailed unpaired Student's t‐test (C)*. ^*^ *p* < 0.05; ^**^ *p *< 0.01; ^***^ *p *< 0.001; ^****^ *p *< 0.0001; ns, no significance.

The three different magnetic field settings (100, 300, and 500 Gs) are shown in **Figure** **3**A. All the magnets used in each of the three magnetic field setups were customized N35 grade cuboid neodymium magnets.

The effects of different concentrations of Ppy‐PCL/Fe_3_O_4_ on the viability of RSC96 cells were assessed using CCK‐8 assays. No significant differences among the groups were observed on day 1. However, on days 3 and 5, although cell viability remained ≈70%, the 1 and 5 wt.% groups showed lower viability compared with PCL (Figure 3B). When the maximum external magnetic field strength (500 Gs) was applied, no significant cytotoxicity was observed in any of the groups compared to the group without a magnetic field. This confirmed that the application of an external magnetic field, either alone or combined with Ppy‐PCL/Fe_3_O_4_, did not affect cell viability (Figure S5, Supporting Information). The cytotoxicity of magnetic nanoparticles has been demonstrated in previous studies.^[^ ^21^ ^]^ Previous studies have shown that magnetic nanoparticles dose‐dependently induced the production of reactive oxygen species (ROS) and disrupted the cytoskeleton.^[^ ^22^, ^23^ ^]^ Although a non‐internalized treatment method was used in the present study, higher magnetic nanoparticle concentrations were still cytotoxic. To balance cytotoxicity and the superparamagnetic effects, it was decided to use the 0.5 wt.% solution in subsequent studies.

Measurement of brain‐derived neurotrophic factor (BDNF) levels by ELISA showed that BDNF levels were higher in the 300 Gs single magnetic field group compared with the 0 and 100 Gs groups. At the same time, the BDNF levels in the 300 Gs group were also higher than those in the 500 Gs group, although the difference was not statistically significant (Figure S6, Supporting Information). When the Ppy‐PCL/Fe_3_O_4_ (0.5 wt.%) were placed in this magnetic field, it was found that the magnetic fiber membrane with the applied magnetic field could also promote BDNF production in RSC96 cells, especially in the 300 Gs group (Figure 3C). The effects of Ppy‐PCL/Fe_3_O_4_ with a static magnetic field on calcium ion influx in RSC96 cells were then evaluated. Measurement of the average fluorescence intensity showed that calcium ion influx increased as the intensity of the magnetic field increased, although, at 300Gs, the influx began to decrease while still exceeding that seen in the 0 Gs group (Figure 3D,E). The Western blotting results showed that a magnetic field of 300 Gs promoted phosphorylation of CREB in RSC96 cells, activating the classical Ca^2+^/p‐CREB/BDNF pathway. These findings suggest the mechanism by which this system upregulated BDNF expression (Figure 3F; Figure S8, Supporting Information).

The effects of magnetic field application on differentiation, the direction of axonal extension, and the length of axonal extension were then examined in PC12 cells with magnetic fields of varying strength and fixed direction. Two orientations of the aligned fibers were used, namely, perpendicular to the magnetic field and parallel to the magnetic field. RSC96 cells were cultured for 3 days under conditions combining Ppy‐PCL/Fe_3_O_4_ with varying magnetic field strengths. The conditioned media were then collected and supplemented with NGF, and subsequently used to treat PC12 cells under the corresponding magnetic field strengths, and the lengths of extended axons were observed (Figure 3G). This showed that the combination of an external magnetic field of 300 Gs, oriented perpendicular to the direction of fiber alignment, resulted in the growth of significantly longer axons (Figure 3H,I). This finding was consistent with the observed increased production of BDNF by Schwann cells under 300 Gs. Western blotting showed that treatment with 300 Gs increased the phosphorylation of TrkB, a receptor of BDNF, and Akt, a critical molecule in the BDNF downstream pathway (Figure 3J; Figure S9, Supporting Information).

The BDNF was first described in the 1980s.^[^ ^24^ ^]^ It is well documented that both exogenous supplementation and endogenous stimulation of BDNF promote the regeneration of peripheral nerves.^[^ ^25^ ^]^ BDNF functions through its interaction with tropomyosin receptor kinase B (TrkB), a cell surface receptor with an intracellular tyrosine kinase domain. Binding to BDNF activates this kinase domain, leading to enhanced autophosphorylation of TrkB and subsequent activation of the PI3K/Akt pathway, promoting neurite outgrowth. The influx of Ca^2+^ ultimately activates the transcription factor, cAMP‐response element binding protein (CREB), or its closely related counterparts, and CREB activation promotes the transcription of BDNF via a Ca^2+^: cAMP‐response element (Ca:CRE) situated in a specific section of the regulatory region of the BDNF gene.^[^ ^26^ ^]^ Additionally, evidence suggests that the observed increase in BDNF mRNA levels in neurons due to the influx of extracellular calcium may result from increased BDNF mRNA transcription, enhanced stability of BDNF mRNA, or a combination of both increased transcription and stability. There has been some research in the field of tissue engineering to accelerate nerve regeneration by increasing BDNF production through an increased influx of calcium ions. Yang et al. described a generalized strategy to integrate photoacoustic (PA) neural stimulation with hydrogel nanocomposite scaffolds to simultaneously increase the influx of calcium ions in the dorsal root ganglion and BDNF expression.^[^ ^27^ ^]^

The present study found that the magnetic nanomaterials influenced the calcium signaling pathway in Schwann cells when stimulated by external magnetic fields, consistent with previous findings. Qin et al. used an external magnetic field combined with NGF‐SPIO‐Au NP to increase calcium ion influx, neuronal differentiation, and the rate of neuroregeneration in PC12 cells.^[^ ^28^ ^]^ Unlike previous studies, the magnetic nanofibers used in the present study showed reduced intracellular uptake, thus effectively controlling cell toxicity.

### 2.3 Ppy‐PCL/Fe_3_O_4_ In Vivo

**Figure 4:**
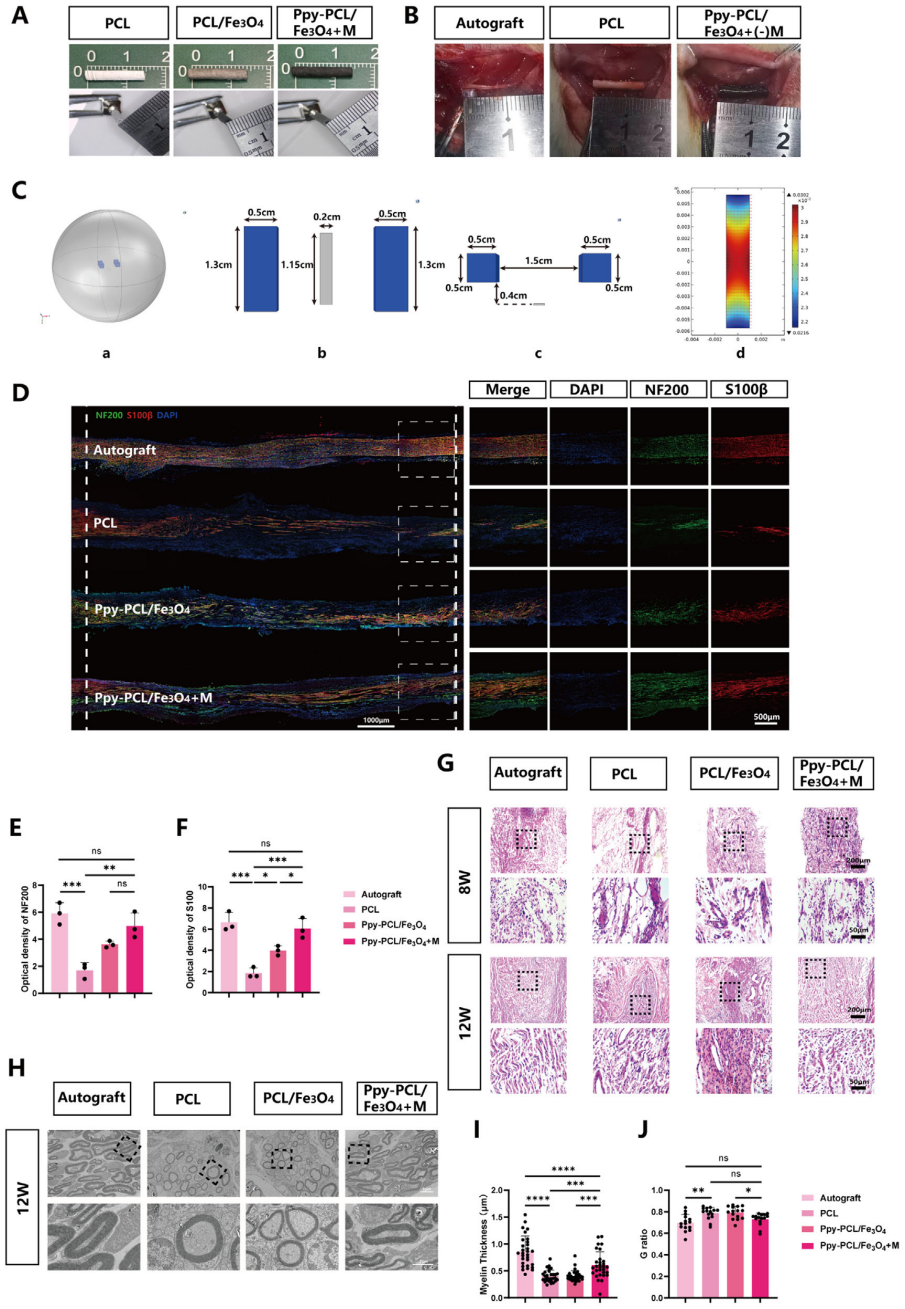
A) Exterior appearance of PCL, PCL/Fe_3_O_4_, and Ppy‐PCL/Fe_3_O_4_: lengths and inner diameters. B) Intraoperative views of implant transplantation in the different groups. C) Modeling analysis of magnetic field strength and direction in the Ppy‐PCL/Fe_3_O_4_ region: a) the spatial position of magnetic field; b) and c) the size of the magnet and its relative position to the conduit area; d) heat map of magnetic field region. D) Images of immunofluorescence staining of longitudinal sections of regenerated nerves in the four groups, showing staining of Schwann cells (S100𝛽), axons (NF‐200), and nuclei (DAPI). E) Quantification of NF‐200 expression, n=3. F) Quantification of S100β expression, n=3. G) Images of H&E‐stained longitudinal sections of regenerated nerves at 8 and 12 weeks post‐surgery in the four groups. H) TEM images of regenerated nerves in the four groups. I)Statistical analysis of myelin sheath thicknesses, n=30. J)Statistical analysis of myelin G ratios, n=15. Statistical analysis was performed using one‐way ANOVA (E, F, I, J). ^*^ *p* < 0.05; ^**^ *p *< 0.01; ^***^ *p *< 0.001; ^****^ *p *< 0.0001; ns, no significance.

Male Sprague–Dawley (SD) rats, aged 8 weeks, were randomly allocated to four groups, namely, the autograft, PCL, Ppy‐PCL/Fe_3_O_4_, and Ppy‐PCL/Fe_3_O_4_ + M groups. The fiber membranes from the different groups were rolled into hollow conduits 14 mm in length and 2 mm in diameter, and the edges were fixed with an 11‐0 suture thread (**Figure** **4**A). One‐centimeter segments of the sciatic nerves in rats from each group were meticulously excised. Groups Ppy‐PCL/Fe_3_O_4_ and Ppy‐PCL/Fe_3_O_4_ + M were treated identically (Figure 4B), while in Group Ppy‐PCL/Fe_3_O_4_ + M, two samarium cobalt magnets were fixed subcutaneously to maintain a magnetic field strength of approximately 300 Gs (Figure 4C). To inhibit reaction to the presence of the magnets, the magnet surfaces were coated with PCL to enhance biocompatibility (Figure S7, Supporting Information). Regeneration in the sciatic nerves was subsequently evaluated at 8 and 12 weeks post‐surgery.

PCL exhibits excellent biocompatibility, good compatibility with organic polymers, and favorable biodegradability. It can be used as a support material for cell growth and is compatible with a variety of conventional plastics. However, despite these favorable properties, PCL is now more commonly used as a base material for biomaterials due to its lack of regenerative capabilities. In the present study, histological analysis of regenerated tissues from the four groups at 8 and 12 weeks was performed, observing the effects of autologous nerve grafts and different types of nerve conduits on peripheral nerve regeneration.

To assess whether the nanoparticles undergo degradation or phagocytosis over time, the hysteresis loop of the nerve conduit implanted for 12 weeks was measured. The results showed that both the superparamagnetism and the saturation magnetization strength at this time were almost identical to those observed immediately after conduit preparation (Figure S11, Supporting Information).

The integrity of axons and myelin sheaths is crucial for signal conduction in the nervous system. Longitudinal sections of regenerating tissue were immunostained with both the central nervous system‐specific proteins β (S100β) and NF‐200 to monitor the extent of remyelination at weeks 8 and 12. As shown in Figure S8 (Supporting Information), at 8 weeks, the autograft group showed significant regeneration of axons and myelin sheaths, while the regenerating tissue in Group Ppy‐PCL/Fe_3_O_4_ + M had reached the proximal end of the defect. However, the nerves in the PCL group had not yet reached the distal end of the defect (Figure S8, Supporting Information). Compared to the 8‐week time point, all four groups showed increased expression of S100β and NF‐200 at 12 weeks, indicating continuous nerve regeneration during the 12‐week period in all groups (Figure 4D). The most distal regions of the regenerating nerves were evaluated in the four groups by measuring the expression of NF‐200 and S100β to assess the levels of axonal and myelin regeneration in each group. The results indicated that at 12 weeks, NF‐200 expression did not differ significantly between the autograft and Ppy‐PCL/ Fe_3_O_4_ + M. After the introduction of an external magnetic field, NF‐200 expression in Group Ppy‐PCL/ Fe_3_O_4_ + M was higher than that in the Ppy‐PCL/Fe_3_O_4_ group but the difference was not statistically significant (Figure 4E). In terms of S100β expression, the autograft and Ppy‐PCL/Fe_3_O_4_ + M groups showed the highest levels, which were significantly higher than those in the other two groups (Figure 4F).

Longitudinal sections of tissue samples from the four groups, stained with hematoxylin and eosin (H&E), demonstrated that at both time points, the PCL and Ppy‐PCL/Fe_3_O_4_ groups showed lower levels of axonal growth and neural cell infiltration, whereas the autograft and Ppy‐PCL/Fe_3_O_4_ + M groups showed greater similarity in these respects (Figure 4G).

The evaluation of regenerated nerve ultrastructure aids in assessing the quality of neural tissue. Transmission electron microscopy (TEM) showed the presence of large clusters of myelinated fibers in the autograft and Ppy‐PCL/Fe_3_O_4_ + M groups, while fine sparse myelinated fibers were seen in tissue from the PCL and Ppy‐PCL/Fe_3_O_4_ groups (Figure 4F). Statistical analysis of the TEM images confirmed that the myelin thickness in Group Ppy‐PCL/ Fe_3_O_4_ + M was greater than that observed in the PCL and Ppy‐PCL/Fe_3_O_4_ groups, although it still lagged behind that in the autograft group (Figure 4I). Furthermore, the calculation of the G‐ratio of the regenerated myelinated fibers to indicate the functionality and structure of the nerve myelin sheaths indicated comparable G‐ratios between the autograft and Ppy‐PCL/Fe_3_O_4_ + M groups (Figure 4J).

**Figure 5:**
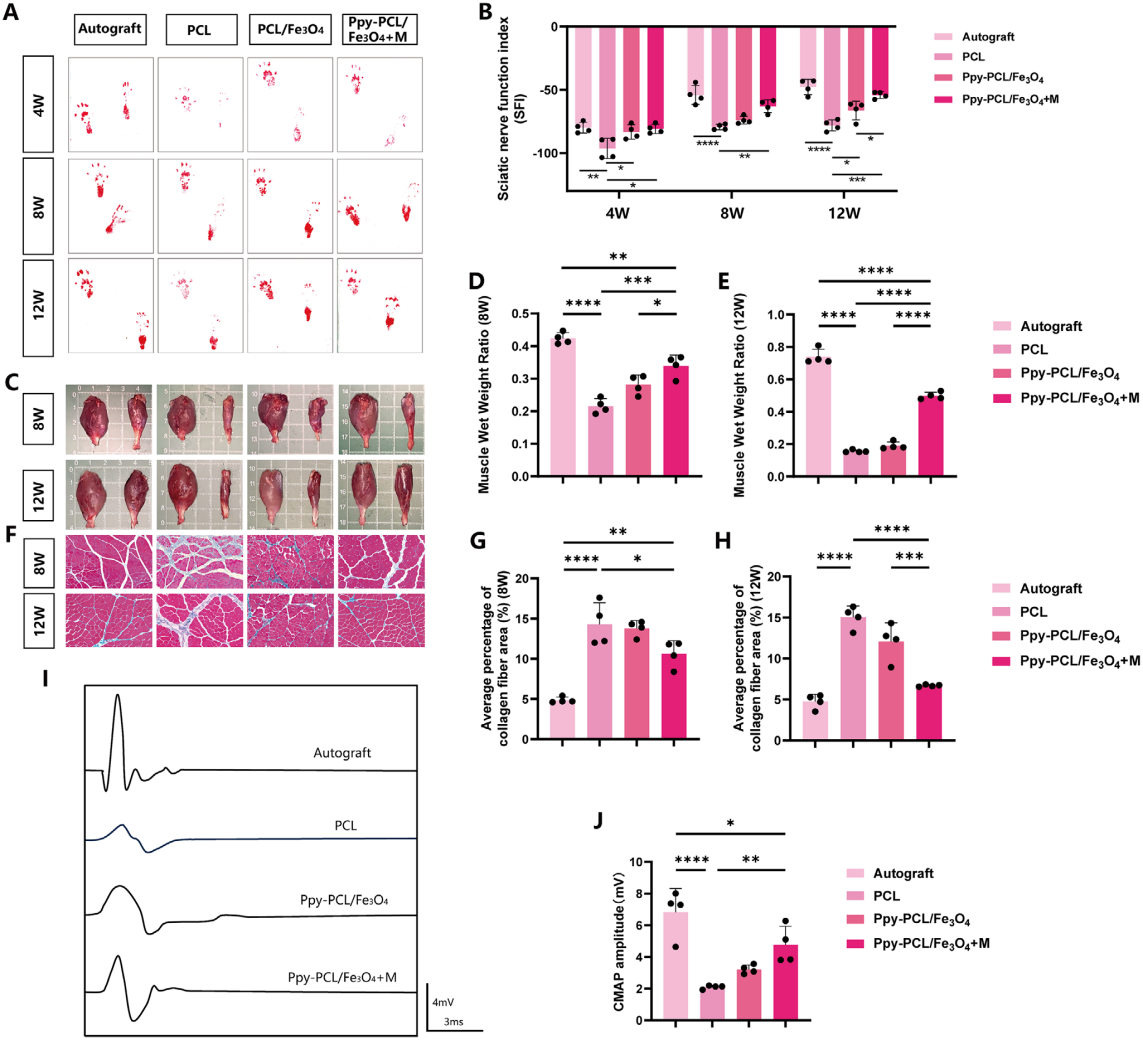
A) Representative images of footprints in the four groups of rats at 4, 8, and 12 weeks after surgery. B) Sciatic nerve function indices (SFIs) in each group at 4, 8, and 12 weeks after surgery, n=4. C) Representative images of harvested gastrocnemius muscles in the four groups. D, E) Weight ratios of gastrocnemius muscles in the different groups at 8 and 12 weeks, n=4. F) Masson trichrome staining of gastrocnemius muscles in each group. G, H) Percentage area of collagen fibers at 8 and 12 weeks (%), n=4. I) Evoked compound muscle action potentials (CMAPs) in each group at 12 weeks after surgery. J) CMAP amplitudes in each group, n=4. Statistical analysis was performed using one‐way ANOVA (B, D, E, G, H, J). ^*^ *p* < 0.05; ^**^ *p *< 0.01; ^***^ *p *< 0.001; ^****^ *p *< 0.0001; ns, no significance.

Functional recovery in the four groups was assessed by analysis of the walking tracks of the animals at 4, 8, and 12 weeks after nerve injury. Analysis of photographs of the hind‐paw tracks and the quantitative measurements of the Sciatic Functional Index (SFI) indicated that functional recovery of the sciatic nerve was least in the PCL group but better in the Ppy‐PCL/Fe_3_O_4_ group (**Figure** **5**A). Comparison of the Ppy‐PCL/Fe_3_O_4_ and Ppy‐PCL/Fe_3_O_4_ + M groups showed no significant difference in their SFI values at 4 weeks; however, at 8 and 12 weeks, the recovery in the Ppy‐PCL/Fe_3_O_4_ + M group was superior to that in the Ppy‐PCL/Fe_3_O_4_ group. No significant differences were seen between the Ppy‐PCL/Fe_3_O_4_ + M and autografts at any of the time points (Figure 5B).

The tissue structures and functioning of target muscles innervated by the damaged nerve are also crucial indicators for assessing the level of nerve regeneration. The gastrocnemius muscle is the terminal target tissue innervated by the sciatic nerve and thus was selected for examination. The atrophy of the gastrocnemius muscle observed in each group at 8 and 12 weeks is shown in Figure 5C. At all measured time points, the autograft group exhibited the best ratio of gastrocnemius muscle wet weight (surgical side/healthy side), followed by the Ppy‐PCL/Fe_3_O_4_ + M group (Figure 5D,E). Notably, significantly better muscle recovery was observed in the autograft and Ppy‐PCL/Fe_3_O_4_ + M groups at 12 weeks relative to that at 8 weeks, whereas the Ppy‐PCL/Fe_3_O_4_ and Ppy‐PCL/Fe_3_O_4_ + M groups showed worse recovery at 12 weeks compared to 8 weeks. It is possible that this was due to muscle atrophy caused by prolonged denervation of the muscle, which might show improvement over time. As shown in Figure 5F–H, the results supported those of the wet weight ratio of the gastrocnemius muscle, with significantly lower proportions of collagen fibers seen in the magnetic field and autologous transplantation groups compared to the other two groups.

Furthermore, electrical signal conduction of the sciatic nerve was examined in each group at 12 weeks (Figure 5I). The compound muscle action potential (CAMP) levels in the autograft group were superior to those in the other groups (Figure 5J), and the electrophysiological performance of groups Ppy‐PCL/ Fe_3_O_4_ and Ppy‐PCL/Fe_3_O_4_ + M was better than that of the PCL group. There was no statistical difference between groups Ppy‐PCL/Fe_3_O_4_ and Ppy‐PCL/Fe_3_O_4_ + M.

**Figure 6:**
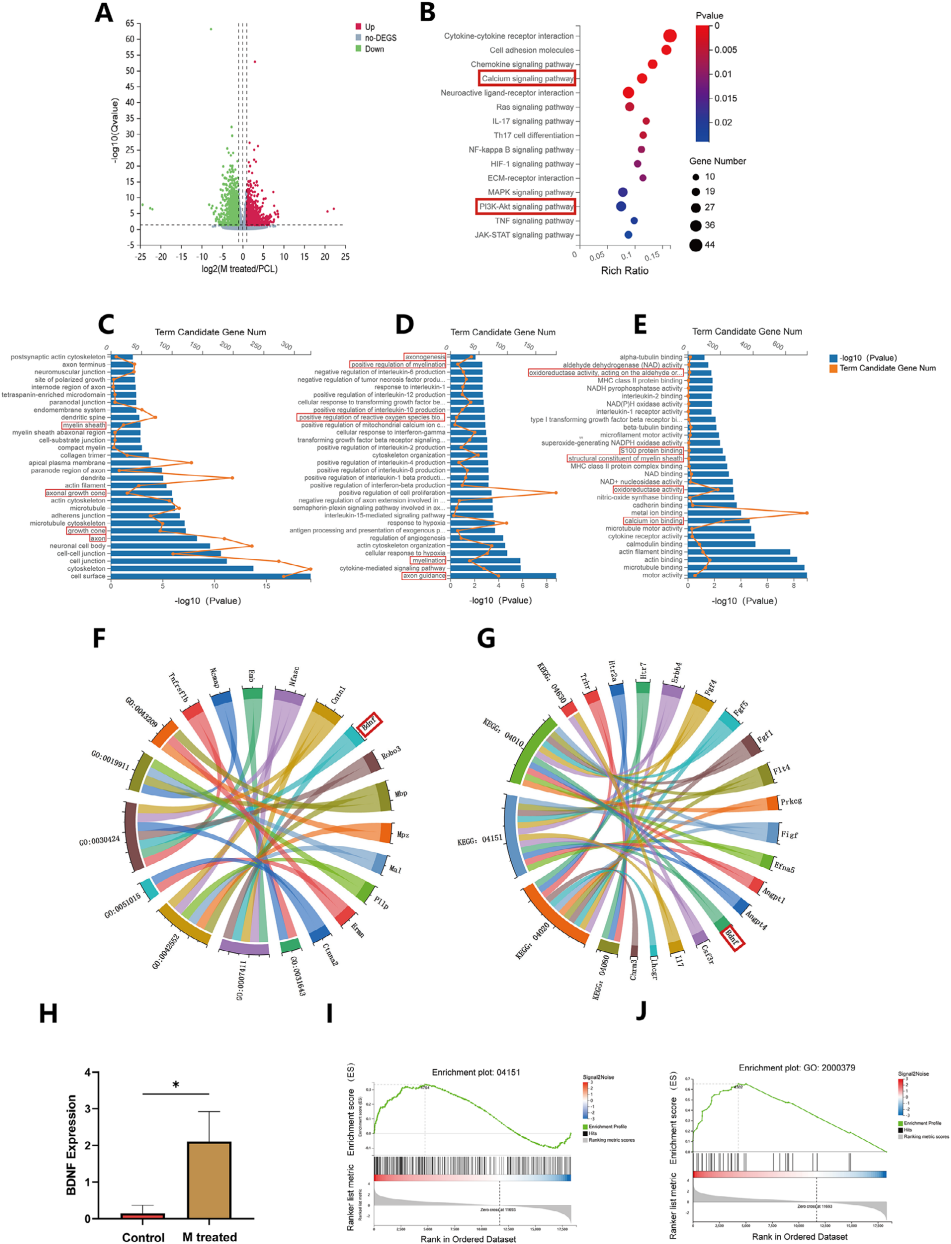
A) Volcano plots showing upregulated (red), downregulated (green), and unchanged (gray) mRNAs between the Ppy‐PCL/Fe_3_O_4_+M and PCL groups. B) KEGG pathways showing significant enrichment of DEGs between the PCL and Ppy‐PCL/Fe3O4+M groups. C, D, E) Gene Ontology (GO) enrichment of DEGs between the PCL and Ppy‐PCL/Fe_3_O_4_+M groups in the biological process (BP), cellular component (CC), and molecular function (MF) categories. F, G) Chordal graphs of KEGG and GO enrichment analyses of DEGs between the PCL and Ppy‐PCL/Fe_3_O_4_+M groups in nerve regeneration‐related pathways. GO: 0043209, myelin sheath; GO: 0030424, axon; GO: 0019911, structural constituent of myelin sheath; GO: 0051015, actin filament binding; GO: 0042552, myelination; GO: 0007411, axon guidance; GO: 0031643, positive regulation of myelination; KEGG: 04630, JAK‐STAT signaling pathway; KEGG: 04010, MAPK signaling pathway; KEGG: 04151, PI3K‐Akt signaling pathway; KEGG: 04020, Calcium signaling pathway; KEGG: 04080, Neuroactive ligand‐receptor interaction. H) BDNF mRNA expression in the PCL and Ppy‐PCL/Fe3O4+M groups, n=3. I) Gene set enrichment analysis (GSEA) of the PI3K‐Akt signaling pathway in the different groups. J) GSEA of positive regulation of ROS metabolism in the different groups.

To elucidate the molecular mechanisms underlying the promotion of nerve regeneration by magnetic fields, RNA sequencing (RNA‐seq) was performed on the sciatic nerves of rats in the PCL and Ppy‐PCL/Fe_3_O_4_ + M groups and identified differentially expressed genes (DEGs). Differential gene expression between the two groups is illustrated in a heatmap (Figure S9, Supporting Information). As shown in the volcano plot (**Figure** **6**A), 1484 genes were upregulated and 1241 genes were downregulated in the Ppy‐PCL/Fe_3_O_4_ + M group relative to the PCL group. KEGG analysis of pathways associated with DEGs highlighted calcium signaling and the PI3K/Akt pathway, a significant downstream pathway of BDNF (Figure 6B). Gene Ontology (GO) analysis showed significant enrichment of DEGs in the cellular component category associated with neural tissues, including axons, growth cones, and myelin. In the GO biological process category, enrichment in processes associated with myelination, axon guidance, and metabolic regulation of reactive oxygen species, among others, was found (Figure 6C,D,E). The interaction network of the GO and KEGG results showed that BDNF was associated with entries such as myelin and axons, as well as with signaling pathways related to nerve regeneration (Figure 6F,G). The mRNA expression levels of BDNF in the experimental group were also significantly higher than those in the control group (Figure 6H). Figure 6I shows the GSEA results of the PI3K/Akt pathway. From these data, it can be observed that magnetic induction activated calcium and the PI3K/Akt pathway, as well as promoting BDNF expression, thereby facilitating axonal guidance and myelin regeneration. Interestingly, the ability of magnetic induction to modulate reactive oxygen species (ROS) production was also observed (Figure 6D,E,J).

**Figure 7:**
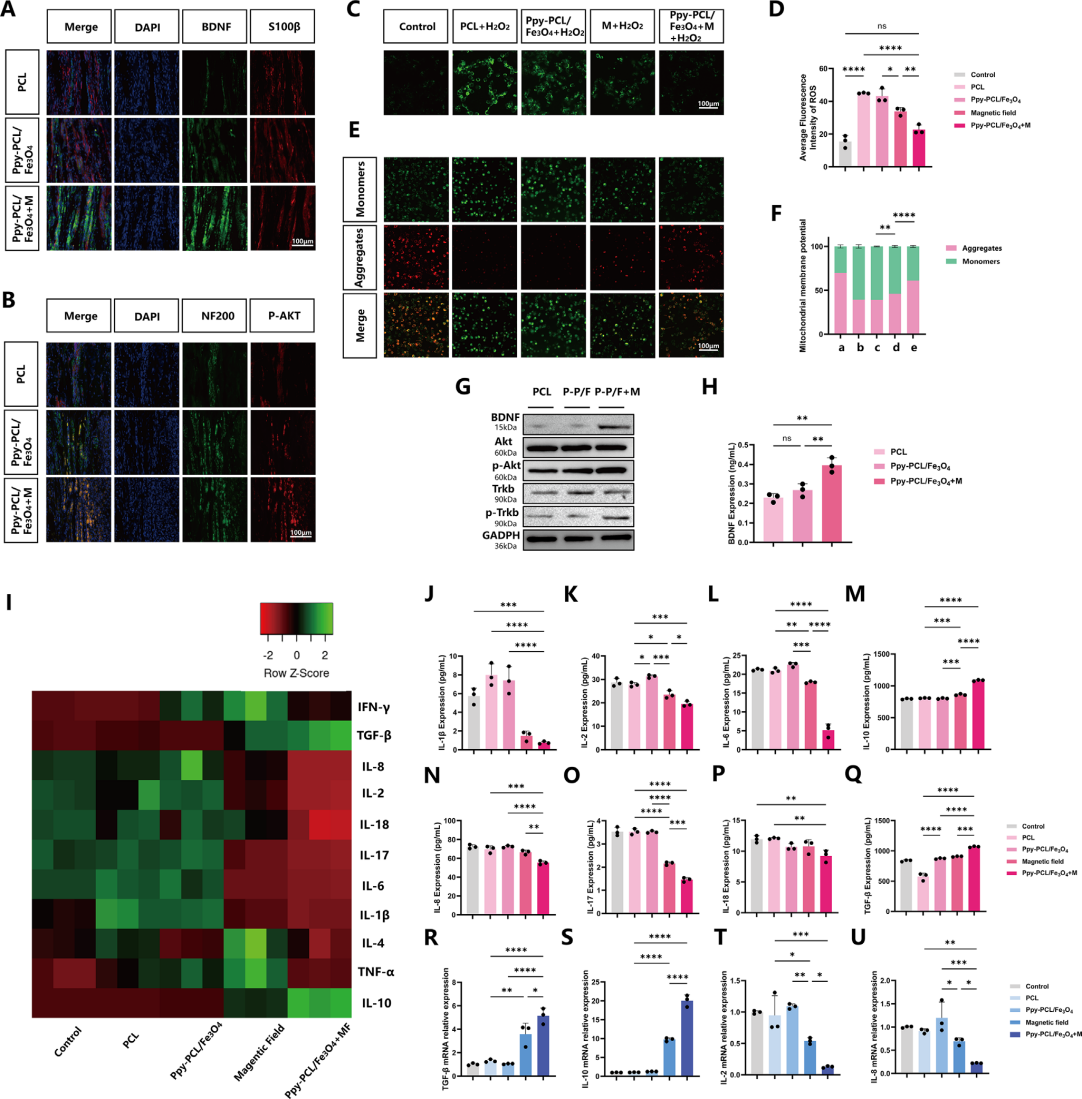
A) Immunofluorescence staining of longitudinal sections of regenerated nerves in the three groups, showing staining of S100𝛽 (red), BDNF (green), and nuclei (DAPI, blue). B) Immunofluorescence staining of longitudinal sections of regenerated nerves in the three groups, showing staining of NF‐200, p‐Akt, and nuclei (DAPI). C Intracellular ROS levels in RSC96 cells were measured by DCF staining. D) Quantitative analysis of DCF fluorescence intensity, n=3. E) Representative images of JC‐1 staining to show mitochondrial membrane potentials by JC‐1 staining. F) Quantitative analysis of the fluorescence intensity of JC‐1, n=4. G) Quantification of Western blotting of BDNF, TrkB, p‐TrkB, Akt, and p‐Akt in the PCL and Ppy‐PCL/Fe_3_O_4_+M groups, n=3. H) Expression of BDNF in the PCL, Ppy‐PCL/Fe_3_O_4_, and Ppy‐PCL/Fe_3_O_4_+M groups in RSC96 cells, as measured by ELISA, n=3. I) Heatmap showing expression of inflammatory factors in RSC96 cells. J‐Q) Expression of IL‐1β, IL‐2, IL‐6, IL‐8, IL‐10, IL‐17, IL‐18, and TGF‐β in RSC96 cells, as measured by ELISA, n=3. R‐U) mRNA expression levels of IL‐2, IL‐8, IL‐10, and TGF‐β, n=3. ^*^ *p* < 0.05, ^**^ *p *< 0.01, ^***^ *p *< 0.001, ^****^ *p *< 0.0001. Statistical analysis was performed using one‐way ANOVA (D, F, H, J, K, L, M, N, O, P, Q, R, S, T, U). ^*^ *p* < 0.05; ^**^ *p *< 0.01; ^***^ *p *< 0.001; and ^****^ *p *< 0.0001; ns, no significance.

The transcriptomic results indicated increased expression of BDNF in the experimental group. Dual immunostaining was performed on longitudinal sections of the regenerated tissue using anti‐S100β and BDNF antibodies. The results showed that the expression of BDNF around the Schwann cells in the Ppy‐PCL/Fe_3_O_4_ + M group was higher than that in the other two groups (**Figure** **7**A). The trends observed in the ELISA measurements of BDNF levels were consistent with those from the immunofluorescence staining (Figure 7H). This suggests that stimulation by external magnetic fields activated BDNF expression by Schwann cells, consistent with the in vitro findings. In addition, dual immunostaining of NF‐200 and p‐Akt was performed on the tissues. The results indicated that Ppy‐PCL/Fe_3_O_4_ + M treatment promoted phosphorylation of Akt in axonal tissues. Notably, despite the absence of magnetic induction in the Ppy‐PCL/Fe_3_O_4_ group, the expression of p‐Akt was higher than that in the PCL group (Figure 7B). To verify the mechanism by which a static magnetic field combined with magnetic nanofibers promoted peripheral nerve regeneration, Western blotting was used to analyze targets suggested by the RNA‐seq results, namely, BDNF, its receptor TrkB, and components of its downstream pathway, PI3K/Akt. The results revealed that the Ppy‐PCL/ Fe_3_O_4_ + M group showed higher expression levels of BDNF compared to the other two groups. Similarly, the expression levels of p‐TrkB and p‐Akt were also higher than those in the other groups (Figure 7G; Figure S14, Supporting Information). This is consistent with the RNA‐seq results.

As the earlier results suggested that Ppy‐PCL/ Fe_3_O_4_ + M treatment may regulate oxidative stress, the association of this treatment with ROS scavenging was investigated in vitro. To simulate ROS‐induced cellular oxidative damage, the antioxidant effects of Ppy‐PCL/Fe_3_O_4_ + M on RSC96 cells treated with 600 µM H_2_O_2_ were evaluated. This showed that treatment with magnetic fields significantly reduced the fluorescence intensity of 2',7'‐Dichlorodihydrofluorescein diacetate (DCF), indicating that the static magnetic field could reduce ROS levels, and Ppy‐PCL/Fe_3_O_4_ could amplify this effect (Figure 7C,D). The JC‐1 staining results are shown in Figure 7E,F. Exposure to the static magnetic field resulted in a reduction in JC‐1 monomers (green) and an increase in JC‐1 aggregates (red) compared to the PCL group. Moreover, the Ppy‐PCL/Fe_3_O_4_ + M group exhibited a more pronounced increase in mitochondrial membrane potentials. Oxidative stress plays a crucial role in the process of neural regeneration. Previous studies have found that excessive accumulation of ROS may delay peripheral nerve regeneration by inducing Schwann cell apoptosis. Researchers have also explored strategies such as the use of melatonin and metformin to reduce ROS production and thus promote neural regeneration.^[^ ^29^, ^30^, ^31^ ^]^ ROS represents a critical regulatory molecule throughout nearly all stages of the inflammatory process. Excessive ROS production by mitochondrial NADPH oxidase, coupled with inadequate compensation by antioxidant systems, can lead to severe cellular and tissue damage, thereby promoting inflammatory responses within the nervous system.^[^ ^32^ ^]^ Here, the levels of various pro‐inflammatory and anti‐inflammatory factors were measured using ELISA in RSC96 cells in the different treatment groups. The results revealed that the magnetic field group and Ppy‐PCL/Fe_3_O_4_ + M group showed increased expression of anti‐inflammatory factors (IL‐10, TGF‐β) together with reduced levels of pro‐inflammatory factors (IL‐1β, IL‐2, IL‐6, IL‐8, IL‐17, IL‐18) (Figure 7J–Q). The RT‐PCR results also indicated that magnetic induction and superparamagnetism stimulated Schwann cells to secrete anti‐inflammatory factors, thereby providing an anti‐inflammatory microenvironment for nerve regeneration (Figure 7I,R–U). In contrast to calcium signaling pathways, the regulation of oxidative stress by static magnetic fields has received little attention. The RNA‐seq results and in vitro experiments in the present study confirmed that the application of magnetic fields alone, as well as in combination with magnetic nanomaterials, has the potential to reduce ROS levels in Schwann cells. This indicates that magnetic systems can modulate the inflammatory microenvironment during nerve regeneration. This finding suggests a broad application potential for magnetic biomaterials in various oxidative stress‐related diseases.

## 3 Conclusion

The present study described the construction of an advanced system predicated on axial guidance via magnetic induction to promote the regeneration of peripheral nerves. This system used a directed external static magnetic field to trigger the superparamagnetic properties of the magnetic nanofibers, operating synergistically with conductive orientation fibers. The primary objective was to provide a novel therapeutic approach for the repair of peripheral nerve injuries. The system optimized the magnetic field intensity to promote calcium influx into Schwann cells, which in turn activated the cAMP response element‐binding protein/brain‐derived neurotrophic factor (CREB/BDNF) signaling pathway. This promoted the secretion of BDNF from the Schwann cells, thereby inducing nerve regeneration. Furthermore, the application of a perpendicular magnetic field, oriented to the axis of the nerve, amplified the biomagnetic effects exerted on the axon, a critical parameter for maximizing the directional guidance of nerve regeneration. Comparative analyses revealed that the regenerative outcomes induced by this system were comparable to the effects of autologous nerve transplantation, underscoring its substantial efficacy. The cumulative evidence presented here underscores the potential of this axial magnetic induction system as a viable alternative for the treatment of extensive nerve defects. This study also has certain limitations. For example, due to the voluntary movements of rats, which can lead to instability in the direction and intensity of the magnetic field, we chose to implant the magnet internally for fixation. This method is clearly not replicable in clinical settings. However, in clinical practice, patient compliance is generally better, and various types of magnetic therapy devices can maintain the stability of the magnetic field. This study provides a foundation for future investigations and potential clinical applications, heralding a new frontier in the field of nerve regeneration therapy.

## 4 Experimental Section

### Materials

All information on materials is listed in Table S1 (Supporting Information).

### Preparation of PCL/Fe_3_O_4_ Fibers

PCL/Fe_3_O_4_ was prepared by electrospinning. Initially, 8 wt.% PCL solutions were prepared in the solvent system of hexafluoroisopropanol (HFIP). The spinning solution was stirred on a magnetic plate at room temperature. Then, different mass fractions of Fe_3_O_4_ (0.1%, 0.5%, 1%, 5%, 20nm) were dispersed into the mixture using an ultrasonic cleaner (Shumei, China). The solution was electrospun using a 5ml syringe paired with a 19G needle at a flow rate of 0.75 mL/h at room temperature. PCL/Fe_3_O_4_ was collected on an aluminium foil collector at the distance of 15 cm from the needle tip with a high voltage of 16 kV.

### Preparation of Ppy‐PCL/ Fe_3_O_4_ Fibers

The preparation of conductive polypyrrole coating on the surface of PCL/Fe_3_O_4_ nanofiber membranes via in situ oxidative polymerization. Prepare stock solutions with a concentration of 2 mol mL^−1^ for pyrrole monomer (Py), ferric chloride (FeCl_3_), and sodium p‐toluenesulfonate (pTS), respectively. Immerse the prepared PCL/Fe_3_O_4_ nanofiber membrane in a 10 mL aqueous solution containing 50 µL of the pyrrole stock solution. Then, add specific volumes of FeCl_3_ and pTS stock solutions in succession, following the volume ratio of Py:FeCl_3_: pTS = 1:2:1, to carry out the oxidative polymerization reaction. The reaction was conducted at a temperature of 4°C for a duration of 24 h. The obtained conductive polypyrrole‐coated nanofiber membrane was sequentially soaked and rinsed with water and anhydrous ethanol to remove unreacted reagents, followed by vacuum drying at room temperature for 12 h.

### Characterization of Ppy‐PCL/ Fe_3_O_4_ Fibers

Functional groups in the nanofiber membrane were identified using a Bruker Vector‐22 spectrometer. The crystal structure of the nanofiber membrane was measured through X‐ray powder diffraction (XRD) using a Rigaku D/Max 2500 diffractometer under Cu Ka radiation (λ = 1.54 Å). The scanning range was from 5° ≤ 2θ ≤ 30°, with a step size of 0.5°, and a scanning rate of 5 s/step. The surface of the materials was observed using a field emission scanning electron microscope (SEM Shimadzu SSX‐550). The average diameter of the fibrous membranes was calculated using Image‐J software. The mechanical properties of the conduit were verified using a universal testing machine with a deformation rate of 10 mm min^−1^. The conductivity (σ) was determined by measuring the resistance (R) of the sample on a smooth surface using a four‐point probe operation (Guangzhou, China). The distance between the probes was 1mm. The resistivity was calculated based on the measured resistance and the thickness of the pellet and Ppy‐PCL/Fe_3_O_4_ fibers, using the following formula:

(1) $$\rho =R\;\times \;F\left(D/S\right)\;\times \;F\left(W/S\right)\;\times \;W\;\times \;Fsp\left(\Omega m\right)$$

where ρ—resistivity, *R*—resistance, *D* — diameter, *S* —probe spacing, *W* —thickness, *Fsp* —probe spacing correction factor, *F*(*D*/*S*) —diameter correction factor, *F*(*W*/*S*) —thickness correction factor.

The reciprocal of the electrical resistance was equal to the electrical conductivity:

(2) $$\sigma =1/\rho \;\;\left(S/m\right)$$

### VSM

To characterize the hysteresis loops of Ppy‐PCL/ Fe_3_O_4_ fibers (0.1%, 0.5%, 1%, 5%), the samples measured by a vibration sample magnetometer (LakeShore, USA). After securing the thin film sample on the sample holder and setting the measurement parameters, a standard sample was used to calibrate the instrument. The sample was then vibrated, and an external magnetic field was applied to measure the sample's magnetization response.

### Cytotoxicity Assay of Ppy‐PCL/ Fe_3_O_4_

Cut the Ppy‐PCL/ Fe_3_O_4_ and PCL fiber into circular pieces (R = 0.3cm). The materials of each group were immersed in 75% ethanol for 24 h and then sterilized under UV irradiation for 2 h. Place PCL and Ppy‐PCL/ Fe_3_O_4_ fibers with different Fe_3_O_4_ concentrations (0.1%, 0.5%, 1%, and 5%) at the bottom of a 96 well plate. Moisten the fiber membrane with ddH_2_O, and air dry under a UV lamp to ensure that the fiber membrane adheres tightly to the bottom of the plate. The RSC96 cells (5 × 103 cells/well, n = 3) were seeded in a 96 well plate covered with nanofibers. Add 100 microliters of culture medium to each well. Add 10 microliters of CCK‐8 (Invigentech, United States) to different fibers at 1, 3, and 5 days, and then the absorbance was measured by (Labserv, China) at 450 nm.

### Magnetic Field Configurations for Cell Cultures

All magnets used in each of the three magnetic field setups were customized cuboid neodymium magnets. The theoretical magnetic flux density as a function of the magnet's position relative to the nanofiber membrane was computed using finite element magnetic field simulation software, Comsol (Stockholm, Sweden). The relative permeability of the two‐dimensional field surrounding the magnet was set to 1.0, which was the relative permeability of air. A color map of the magnetic flux density at specific locations on the membrane was generated using the values obtained from the simulation.

### Ca^2+^ Flow Cytometry

Cut the Ppy‐PCL/ Fe_3_O_4_ (Fe_3_O_4_ wt.% = 0.5%) fibers into a circular shape (R = 1cm). Disinfection steps were the same as 2.1. Place the nanofiber membrane at the bottom of the culture dish, then the RSC96 cells were seeded on the surface of the fiber membrane. Place the culture dish in a static magnetic field with a center point of approximately 0, 100, 300, 500Gs. After 24 h of incubation, the cells were trypsinized and harvested, followed by measurement of Ca^2+^ influx levels using the EZCell Calcium Detection Kit (Biovision, United States) according to the manufacturer's protocol. Measurement of intracellular calcium indicator fluorescence intensity by flow cytometry showed intracellular Ca^2+^ levels.

### ELISA

The preparation of fibers and cell seeding is the same as described in Section 2.3.

The levels of index were measured in triplicate using ELISA kits (Table S1, Supporting Information) according to the manufacturer's instructions. Absorbance was measured at 450 nm with a microplate reader (BioTeK, Vermont, United States).

### PC12 Cell Differentiation

The bioactivity of BDNF, secreted by RSC96 cells on Ppy‐PCL/Fe_3_O_4_ scaffolds combined with a static magnetic field, was assessed through the differentiation of PC12 cells. Initially, PC12 cells (2.0 × 10^6 cells/well) were plated in 3.5 cm glass dishes and incubated for 24 h. Subsequently, the medium was replaced with conditioned medium harvested from RSC96 cells cultured on scaffolds for three days (with a medium change on the second day). This conditioned medium was then mixed with fresh complete medium at a 1:1 ratio, which was then supplemented with 2.5% FBS. Cultures maintained in standard medium served as controls. After a three‐day culture period, the differentiated PC12 cells were fixed using 4% paraformaldehyde and stained for their cytoskeleton with phalloidin (Solarbio, China) and for their nuclei with DAPI (Beyotime, China). Observations of the stained cells were made using confocal microscopy. The lengths of the neurites was quantified using Image J software.

### Western Blot

Protein samples were prepared using RIPA lysis buffer (Servicebio, China), separated by SDS‐PAGE, and transferred onto PVDF membranes. After blocking with 5% BSA, the membranes were incubated with anti‐Trkb (Servicebio, GB11295‐1), P‐Trkb (Abcam, ab229908), AKT (Servicebio, GB111114), p‐AKT (Servicebio, GB150002), BDNF (Abcam, ab205067), CREB (Servicebio, GB111052), and p‐CREB (Servicebio, GB114322) antibodies. Protein bands were visualized using an enhanced chemiluminescence detection method (Tanon, 180‐506) with Tanon (4600) imaging.

### Animals and Surgical Procedures

Forty‐eight male Sprague‐Dawley (SD) rats, aged 2 months and weighing between 200 and 250 g, were acquired from Changsheng Biotechnology (Liaoning, China). The execution of all animal experiments adhered stringently to the guidelines outlined in the US National Institutes of Health (NIH) Guide for the Care and Use of Laboratory Animals, as established by the US National Academy of Sciences, with approval granted by the Jilin University Administration Committee of Experimental Animals and Changchun Weishi Testing Technology Service Co. (IACUC Issue No.: 20221117‐01). These rats were randomly allocated into four experimental cohorts: autograft, PCL nerve conduit, Ppy‐PCL/Fe_3_O_4_, and Ppy‐PCL/Fe_3_O_4_ combined with a magnetic field. Under general anesthesia induced by ketamine (50 mg kg−1 body weight), each rat underwent microsurgical procedures conducted by a singularly skilled surgeon. The initial step involved the preparation of the rat, including sterilization of the surgical area on the lateral aspect of the right thigh (marked by a roughly 4 cm incision aligned parallel to the lower margin of the femur), extending to the right side of the hip and thigh. An incision of approximately 4 cm was made on the lateral side of the right thigh to meticulously expose the sciatic nerve, with the entire surgical process being facilitated by a surgical microscope.

In the autograft group, a 10 mm segment of the sciatic nerve was surgically removed, inverted, and reinserted. The autografts were then anchored to the epineurium of both the proximal and distal nerve ends using an 8‐0 monofilament nylon suture. For the groups designated as PCL nerve conduit, Ppy‐PCL/Fe_3_O_4_, and Ppy‐PCL/Fe_3_O_4_ with an added magnetic field, the sciatic nerve's proximal end was severed first. Subsequently, the nerve stumps were repositioned 1 mm towards the proximal end of a 14‐mm conduit, resulting in a 10 mm interstice. These were then secured with a single 10‐0 nylon suture. Specifically, for the Ppy‐PCL/Fe_3_O_4_ with magnetic field group, two samarium cobalt magnets were implanted subcutaneously following the suturing of the Ppy‐PCL/Fe_3_O_4_ at the site of injury. The spacing between the magnets and the conduit was determined based on models from Comsol, with the conduit being stitched to adjacent muscles to ensure its positional stability. This step was replicated at the nerve's distal segment. The conduits, with a diameter approximating 2 mm, were matched to the diameter of the sciatic nerve. The surgical incisions were finally closed using 4‐0 silk sutures.

### Walking Track Analysis

Walking track analysis was performed at 8 and 12 weeks post‐operatively to assess motor recovery among the groups. This involved staining the hind limbs of the rats with red ink and then encouraging them to traverse a box measuring 90 cm by 13 cm by 20 cm, at the bottom of which lay a sheet of white paper. The Sciatic Function Index (SFI) was determined by analyzing various parameters of the rats' footprints, employing the subsequent formula:

(3) $$\begin{array}{cc} SFI & =109.5\times \left(ETS-NTS\right)/NTS-38.3\times \left(EPL-NPL\right)/NPL\\ & \;+13.3\times \left(EIT-NII\right)/NIT-8.8 \end{array}$$

Toe spread (TS) was the distance between the 1st and 5th toes, inter‐toe spread (IT) was the distance between the 2nd and 4th toes, and PL was the length of the footprint. The E in the formula indicates the experimental or injured side while the N indicates the normal or uninjured side.

### Electrophysiological Assessment

At 12 weeks post‐operation, the rats underwent anesthesia for the evaluation of motor nerve function on the injured side using electromyography (Haishenyidian, China). For this procedure, a stimulating electrode was positioned at the proximal and distal locations of the injured nerve in the male rats, and a recording electrode was placed on the gastrocnemius muscle. Compound Muscle Action Potentials (CMAPs) for the rats across the different experimental groups were recorded following electrical stimulation. Corresponding peak amplitudes of the CMAPs were then calculated.

### Histological Analysis of Regenerated Nerve Tissues

At the 8 and 12‐week post‐operative marks, Sprague‐Dawley rats were euthanized for the retrieval of nerves from the experimental side (n = 6). The nerves were fixed in 4% paraformaldehyde, freshly prepared, for 16 h, followed by a 24‐hour immersion in 30% sucrose. After another 24‐hour sucrose immersion, the tissues were encapsulated in O.C.T. Compound and instantly frozen with liquid nitrogen. Using a Leica cryostat microtome (Wetzlar, Germany), the samples were longitudinally sectioned into 12 µm slices. For Hematoxylin and Eosin (H&E) staining, the longitudinal sections underwent methanol fixation and were rinsed twice with deionized (DI) water, then stained with Hematoxylin for 3.5 minutes. In the immunofluorescent staining protocol, three rats from each group were administered a lethal dose of pentobarbital (25%, intraperitoneal injection) at the 8 and 12‐week post‐operative intervals. The nerve grafts were subsequently collected and longitudinally sliced into 12µm sections using a cryostat. Axons and myelin sheaths were stained with anti‐neurofilament NF200 antibody (1:80; Sigma‐Aldrich, Germany) and S100b antibody (1:200; Abcam, Cambridge, UK), followed by the application of fluorescently labeled secondary antibodies (Invitrogen, 1:500). These dual‐stained sections were examined using fluorescence microscope (Nikon, Japan).

### TEM

In summary, the sciatic nerve underwent fixation in 2.5% glutaraldehyde at 4°C for a duration of 24 h. This was followed by a rinse in phosphate‐buffered saline for 10 minutes. The nerve was then dehydrated through a progressive series of alcohol concentrations, ultimately being encapsulated in epoxy resin. Ultrathin slices, ranging from 50 to 80 nm, were produced, then subjected to staining with uranyl acetate and lead citrate. Observation of the axons and myelin sheaths was conducted using Transmission Electron Microscopy (TEM) on a FEI TECNAI‐SPIRIT instrument.

### Gastrocnemius Muscle Evaluation

Rats were euthanized at 8 and 12 weeks post‐operation, and gastrocnemius muscles from both the healthy and injured sides were harvested and weighed to determine the weight ratio. The samples were then fixed in a 4% paraformaldehyde solution for 24 h. Following dehydration and embedding in paraffin, the muscles were longitudinally sectioned to produce 5 µm slices, which were then subjected to Masson's trichrome staining. Measurements were taken of the cross‐sectional area of the muscle fibers.

### mRNA‐sequencing and data analysis

RNA was extracted from sciatic nerve tissue treated with PCL nerve conduits and Ppy‐PCL/Fe_3_O_4_ under magnetic field, after homogenization, lysis, and purification. The raw sequencing data were filtered using SOAPnuke (v1.5.6). The clean data were aligned to the reference genome using HISAT2 (v2.1.0) and to the reference gene set using Bowtie2 (v2.3.4.3). Gene expression quantification was performed with RSEM (v1.3.1), and a clustering heatmap of gene expression across different samples was drawn using pheatmap (v1.0.8). Differential gene expression analysis was conducted using DESeq2 (v1.4.5) with conditions set to Q value ≤ 0.05. To further explore gene functions related to phenotypic changes, GO, KEGG, and GSEA enrichment analyses of differentially expressed genes were performed based on the hypergeometric test using Phyper, with a threshold of P value ≤ 0.05, defining significant enrichment among the candidate genes.

### In Vitro Protection From Oxidative Stress Induced by H_2_O_2_

RSC96 cells were exposed to 600 µM hydrogen peroxide for 24 h. The levels of reactive oxygen species (ROS) in RSC96 cells were quantitatively analyzed by measuring the fluorescence intensity of 2ʹ,7ʹ‐dichlorofluorescin diacetate (DCFH‐DA) (D6883, Sigma) using confocal microscopy.

### Mitochondrial Membrane Potential Detection

Mitochondrial membrane potential (MMP) was assessed using a JC‐1 staining kit, strictly following the manufacturer's instructions. RSC96 cells were exposed to 600 µM hydrogen peroxide for 24 h, followed by the addition of JC‐1 staining working solution. The cells were then incubated for 30 minutes at 37°C in a dark incubator. After incubation, the cells were washed three times with JC‐1 buffer solution. Finally, observations and image acquisition were performed using a confocal microscope. The fluorescence intensity of representative images was quantitatively analyzed using ImageJ software.

### Real‐time quantitative PCR analysis

The expression of inflammation‐related genes (IFN‐γ, TGF‐β, TNF‐α, IL‐1β, IL‐2, IL‐4, IL‐6, IL‐8, IL‐10, IL‐17, and IL‐18) was detected using qRT‐PCR. Total RNA was extracted from different groups using the Trizol method (Thermo Fisher Scientific, USA). Quantitative RT‐PCR was performed using SYBR GreenER qPCR Mix and gene‐specific primers. The standard PCR conditions were 30 s at 95°C, followed by 5 s at 95°C and 34 s at 60°C, for a total of 40 cycles. Relative expression was calculated using the 2‐ΔΔCT method.

### Statistical analysis

Statistical analyses were performed utilizing GraphPad Prism version 8.0 (CA, USA) and Origin version 9.0 (Northampton, USA). Data were represented as mean ± standard deviation (SD). For the analysis of differences within multiple groups, one‐way ANOVA testing followed by a Tukey post‐hoc test was employed, while comparisons between two groups were conducted using a two‐tailed unpaired Student's t‐test. At least 3 independent experiments were performed. A P‐value less than 0.05 was deemed to indicate statistical significance (^*^ *p* < 0.05; ^**^ *p* < 0.01; ^***^ *p* < 0.001; ^****^ *p* < 0.0001; ns, no significance).

## Conflict of Interest

The authors declare no conflict of interest.

## Supporting information



Supporting Information

## Data Availability

The data that support the findings of this study are available from the corresponding author upon reasonable request.
