# Supplementary material for: Aligned Conductive Magnetic Nanofibers with Directional Magnetic Field Stimulation Promotes Peripheral Nerve Regeneration
Source: Adv Sci (Weinh). 2025 Jul 6;12(37):e01665. doi: 10.1002/advs.202501665 (PMC12499492; doi:10.1002/advs.202501665)
Supplement: Supplementary file 1 — Supporting Information [file ADVS-12-e01665-s001.docx]

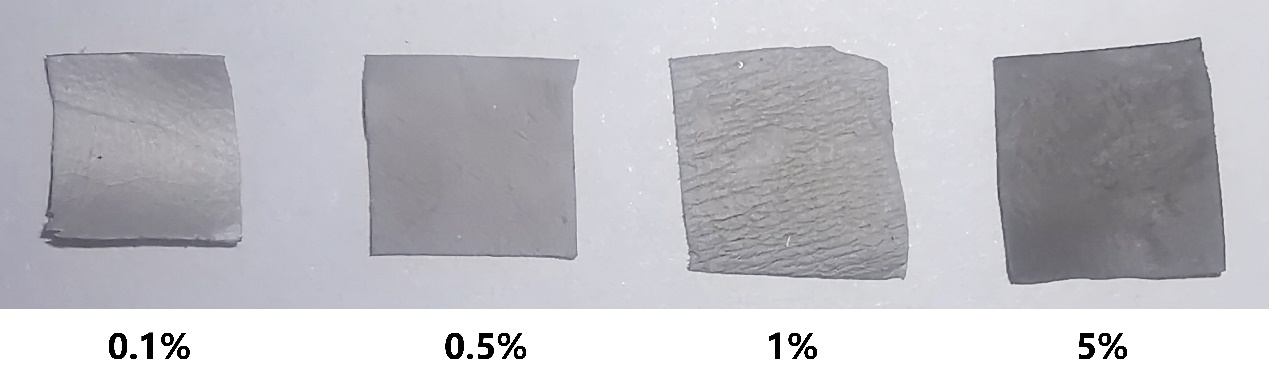


**Supplementary Figure 1. Photographs of PCL/Fe3O4 magnetic nanoparticle fibers at various concentrations (0.1, 0.5, 1, and 5 wt%)**


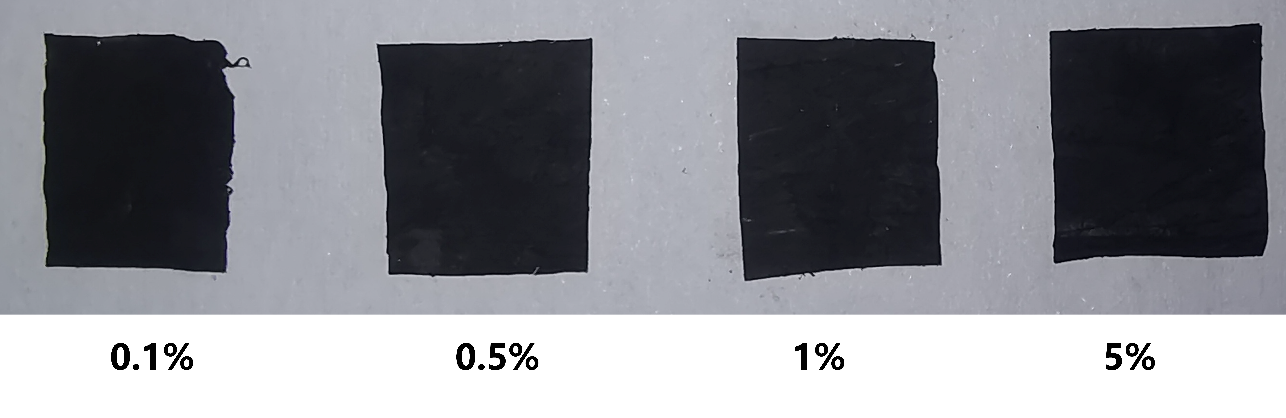


**Supplementary Figure 2. Photographs of Ppy-PCL/Fe3O4 magnetic nanoparticle fibers at various concentrations (0.1, 0.5, 1, and 5 wt%)**


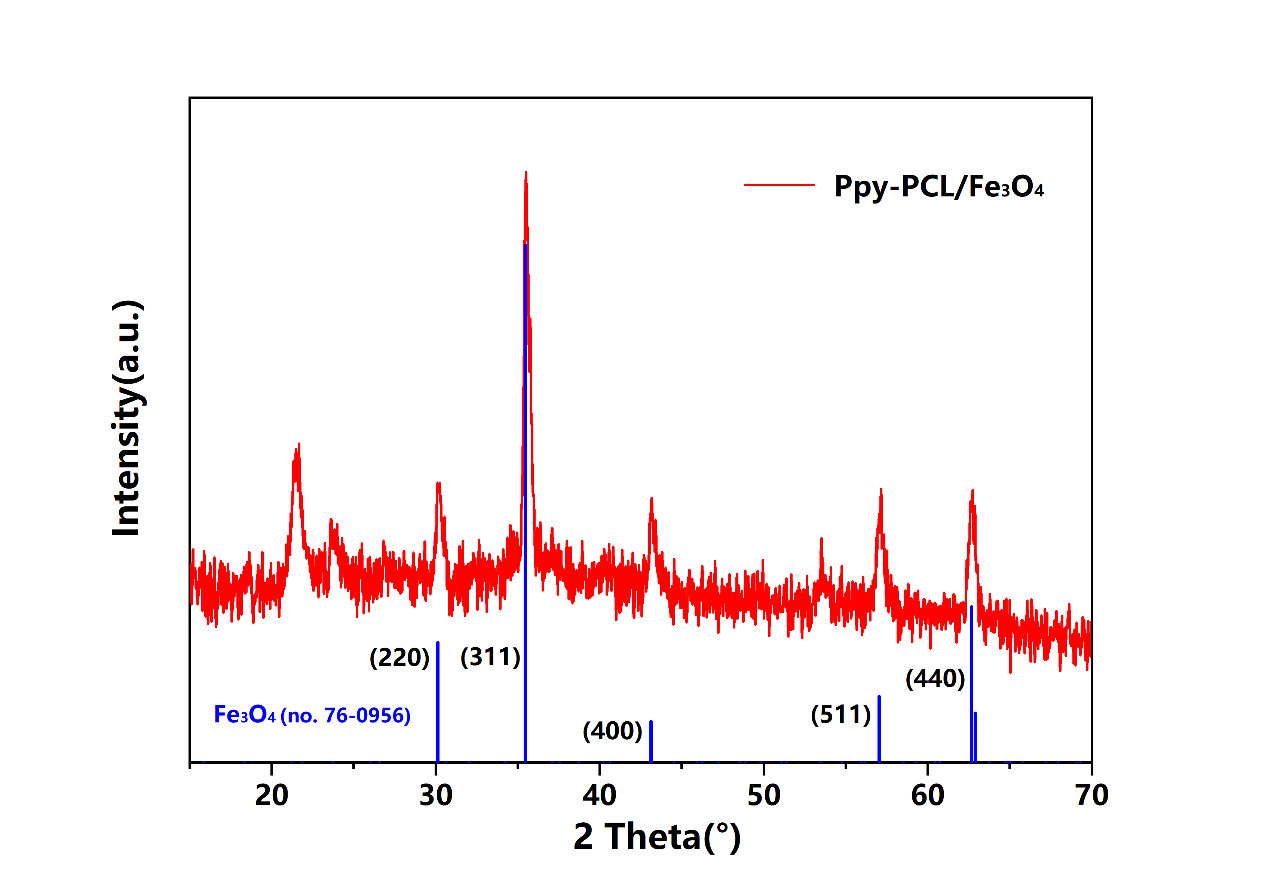


**Supplementary Figure 3. XRD pattern of Ppy-PCL/Fe3O4 nanofibers**


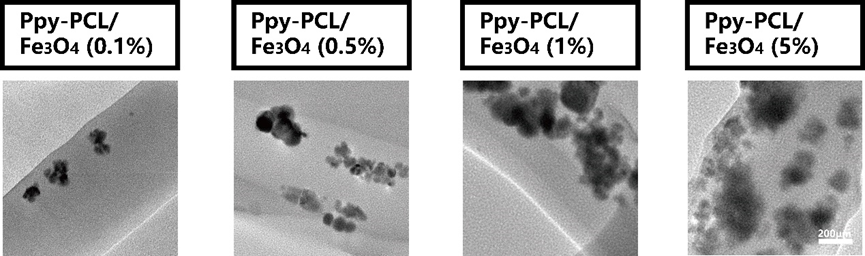


**Supplementary Figure 4. Transmission electron microscopy images of the fiber membranes**

**Supplementary Figure 5. The conductivity of varying concentrations of Ppy-PCL/ Fe3O4 under the stimulation of a 500 Gauss static magnetic field**

**Supplementary Figure 6. Cell viability of RSC96 cells treated with Ppy-PCL/ Fe_3_O_4_ combined with a 500 Gauss static magnetic field**

**Supplementary Figure 7. Levels of BDNF in the different magnetic fields were measured by ELISA**

**
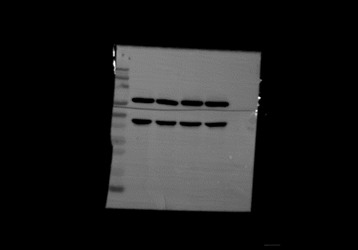
**
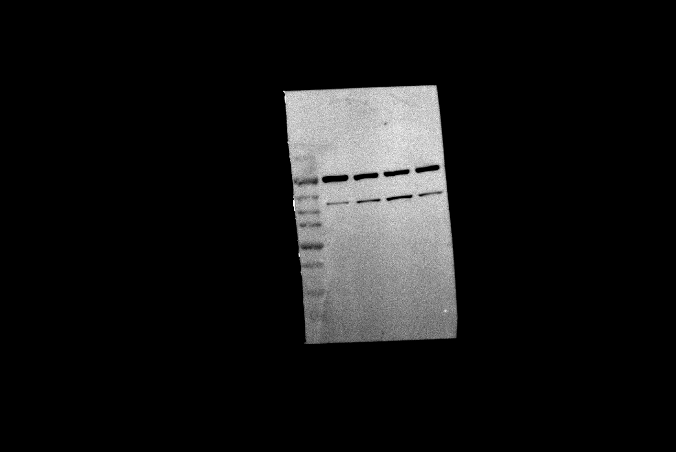


**Supplementary Figure 8. Original image of Figure 3F**


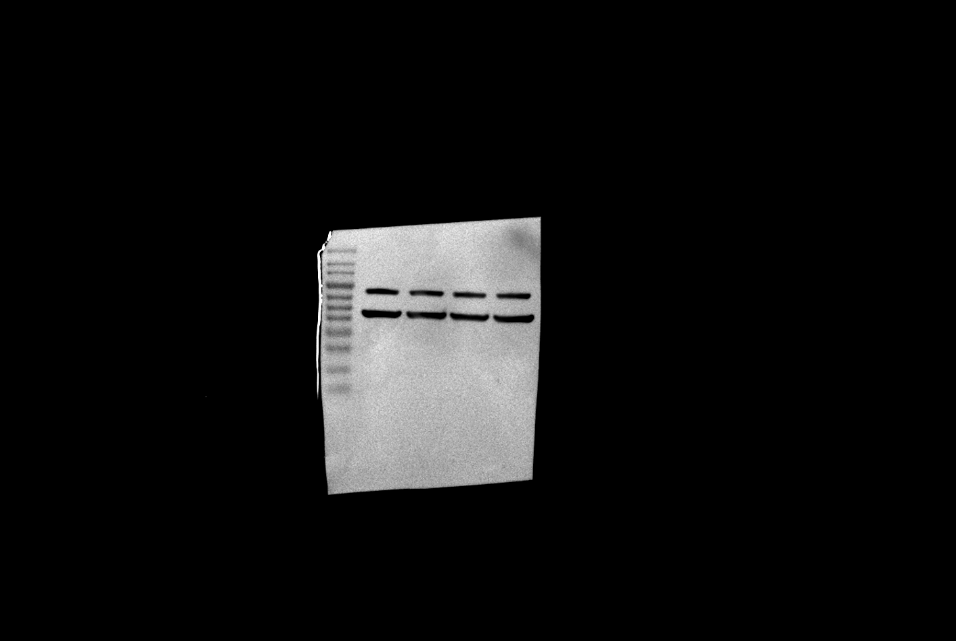

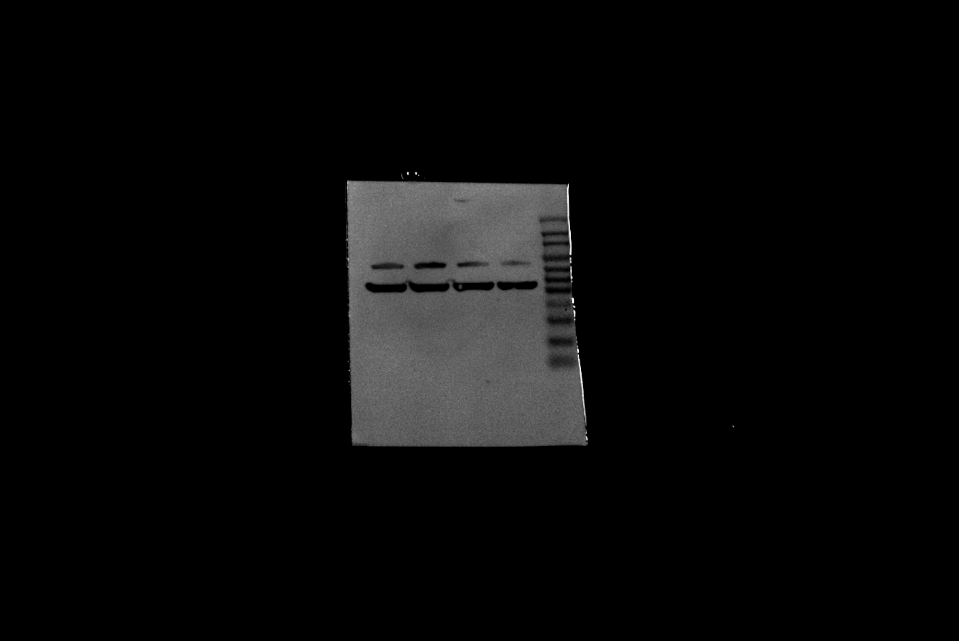


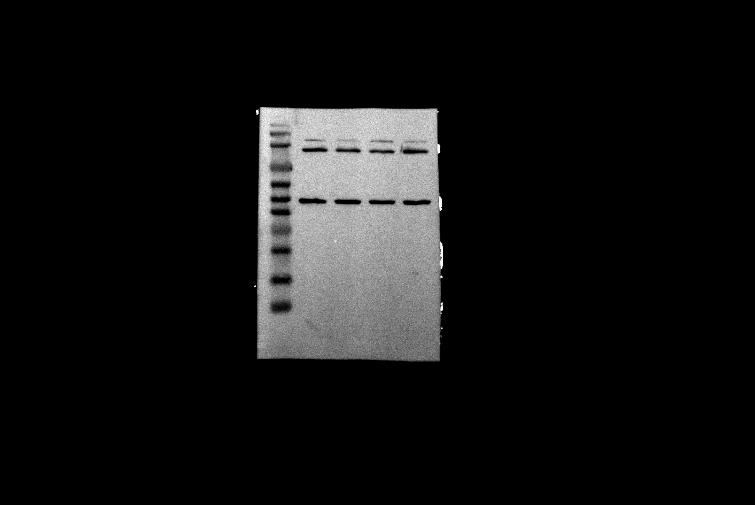

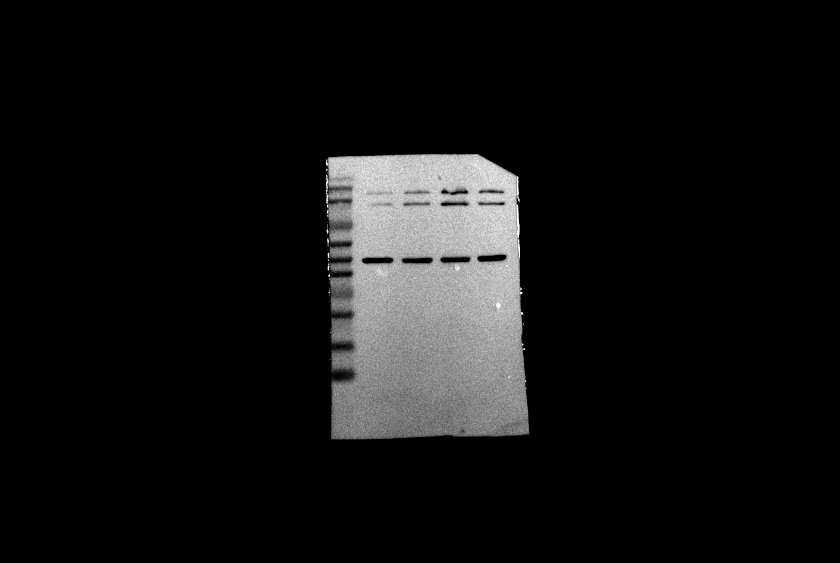


**Supplementary Figure 9. Original image of Figure 3J**

**
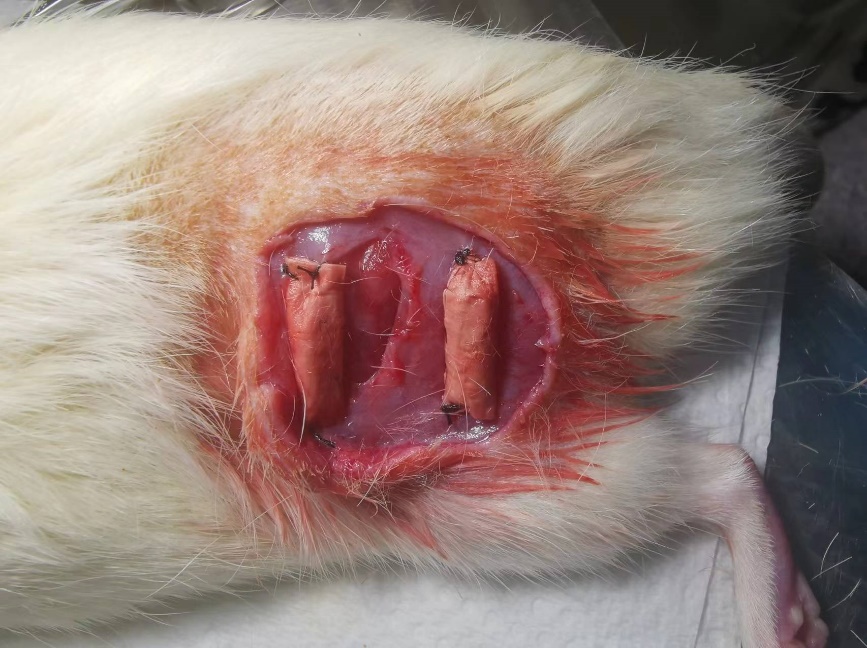
**

**Supplementary Figure 10. Photographs of magnets covered with PCL**


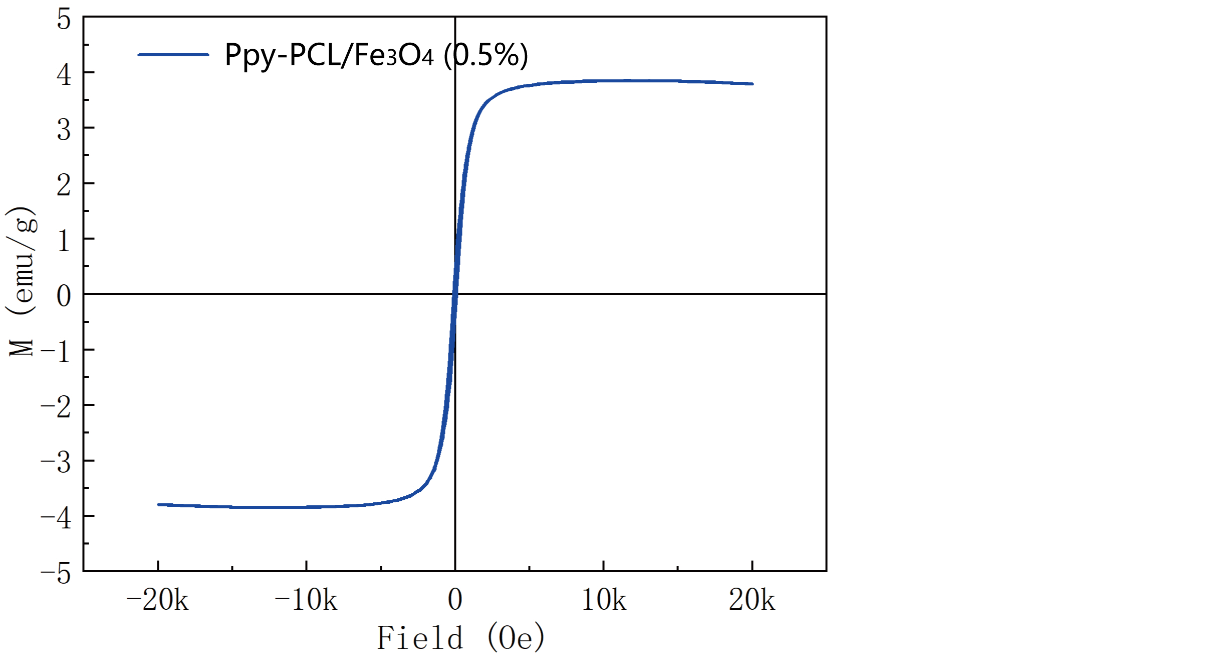


**Supplementary Figure 11. Hysteresis loop analysis of Ppy-PCL/ Fe_3_O_4_ (0.5%) after 12 weeks of in vivo cultivation**


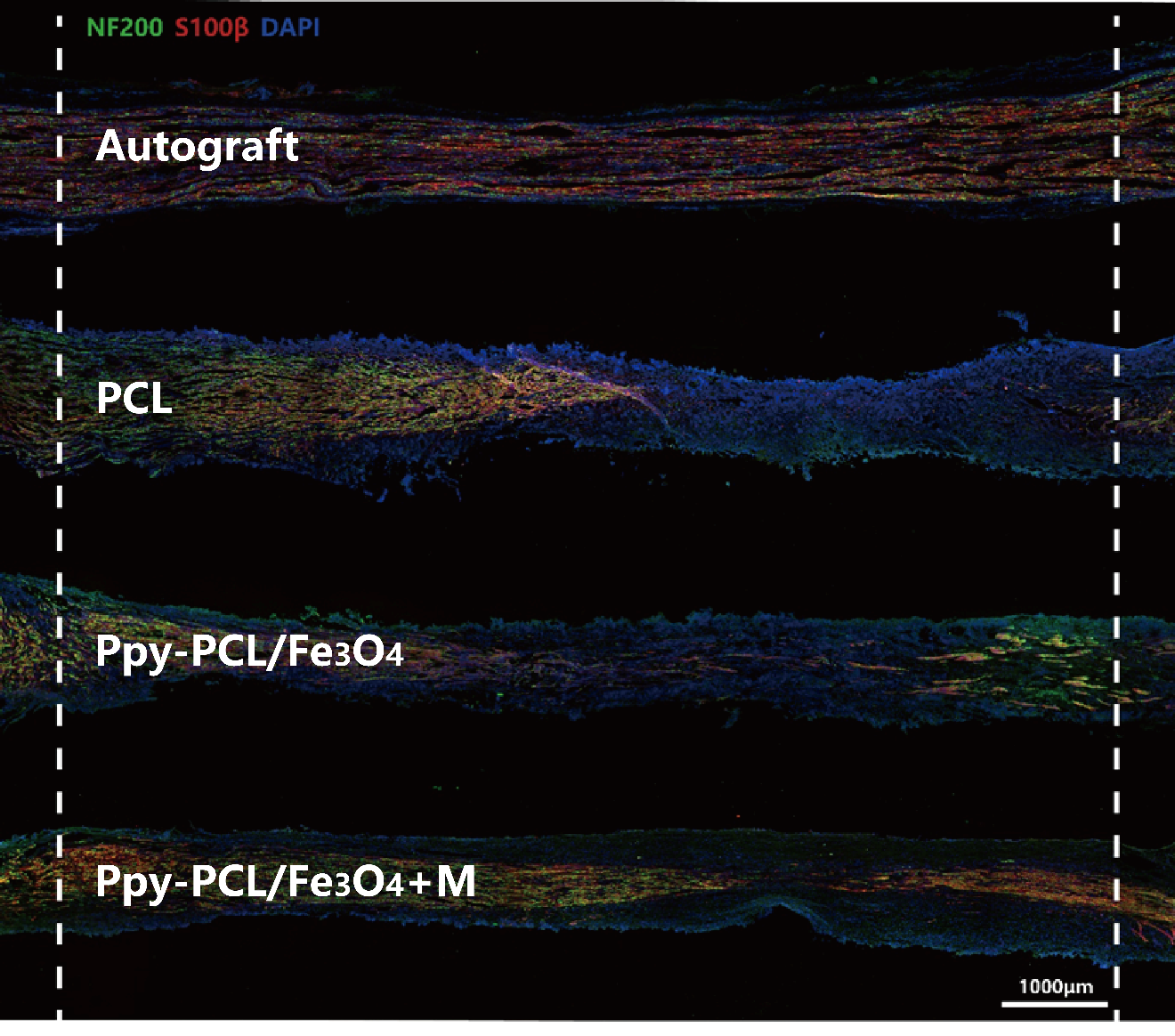


**Supplementary Figure 12. At 8 weeks post-operation, longitudinal sections of regenerating nerves from the four groups were captured in immunofluorescence images. Schwann cells (S100β), axons (NF-200), and cell nuclei were stained respectively**


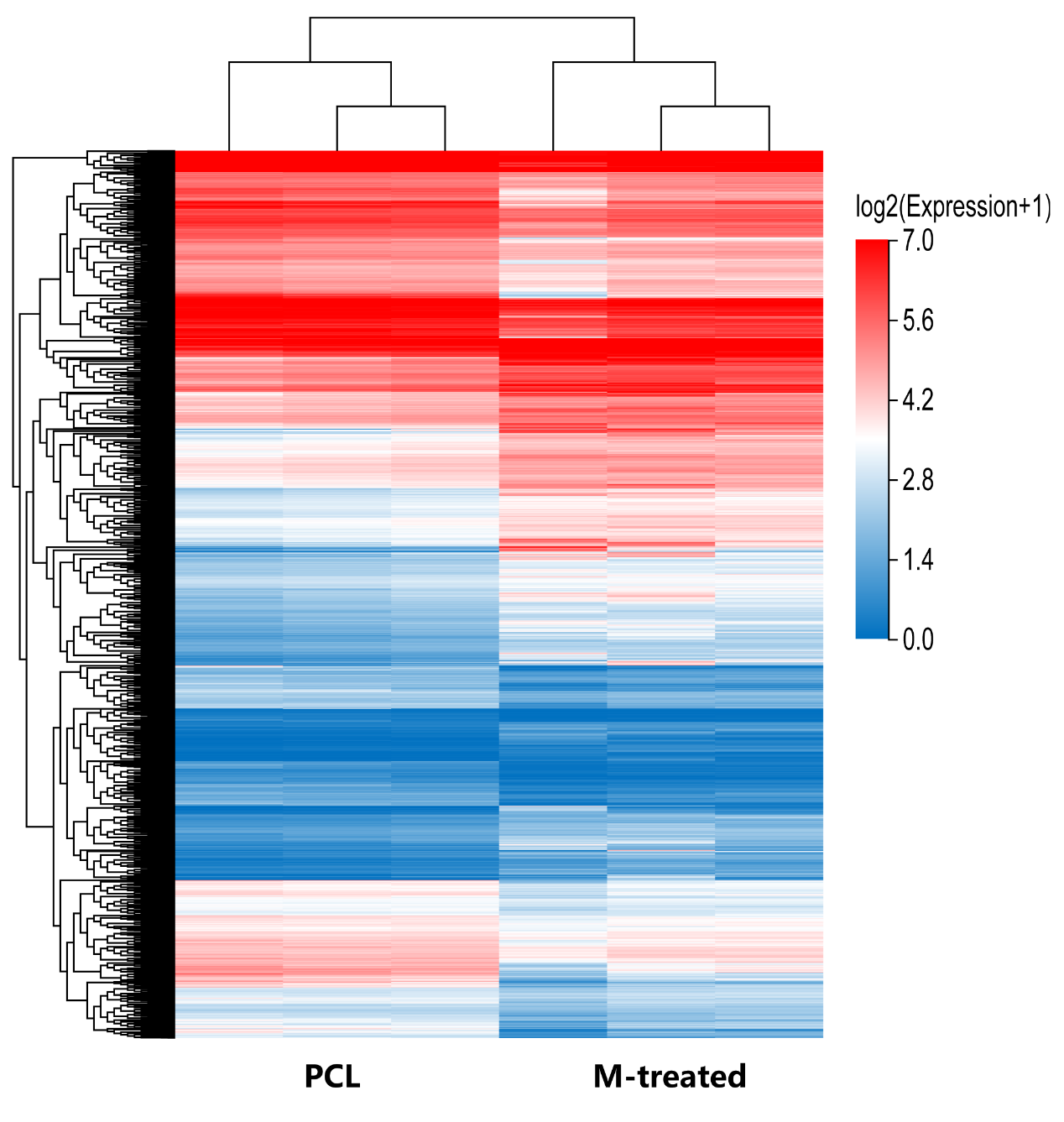


**Supplementary Figure 13. Gene expression heatmap of PCL and** **Ppy-PCL/Fe_3_O_4_ groups (n = 3)**


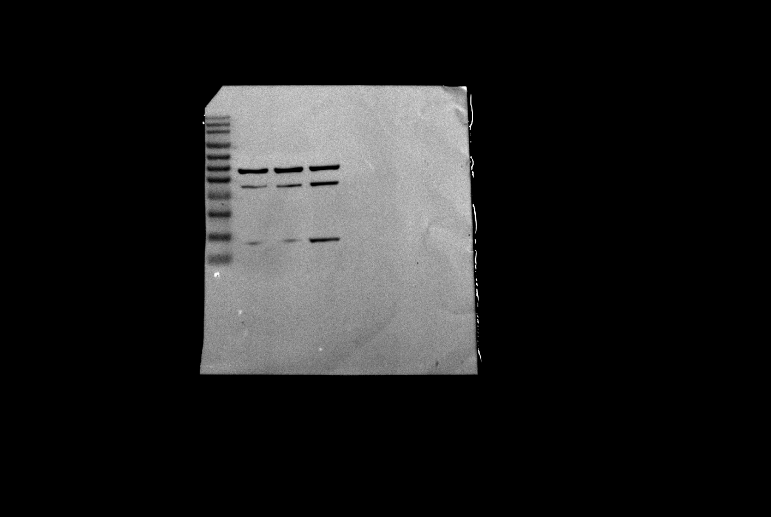

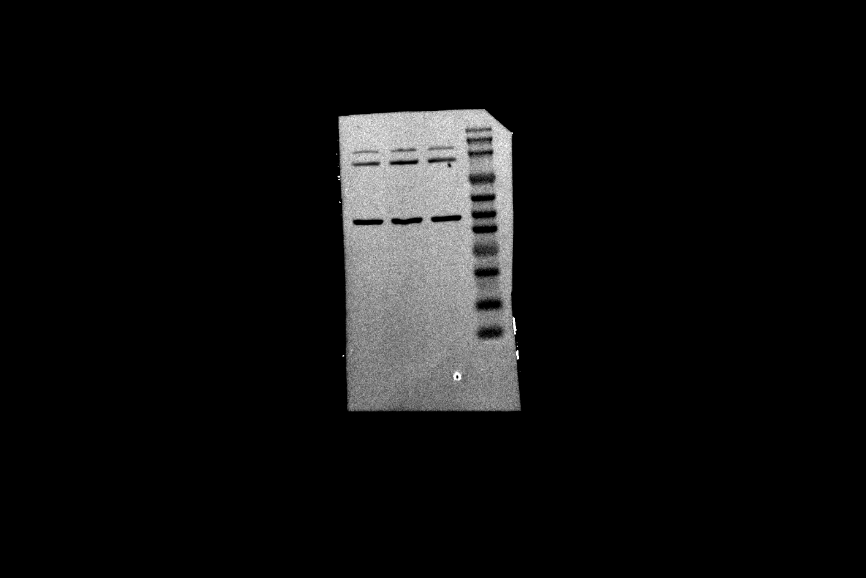


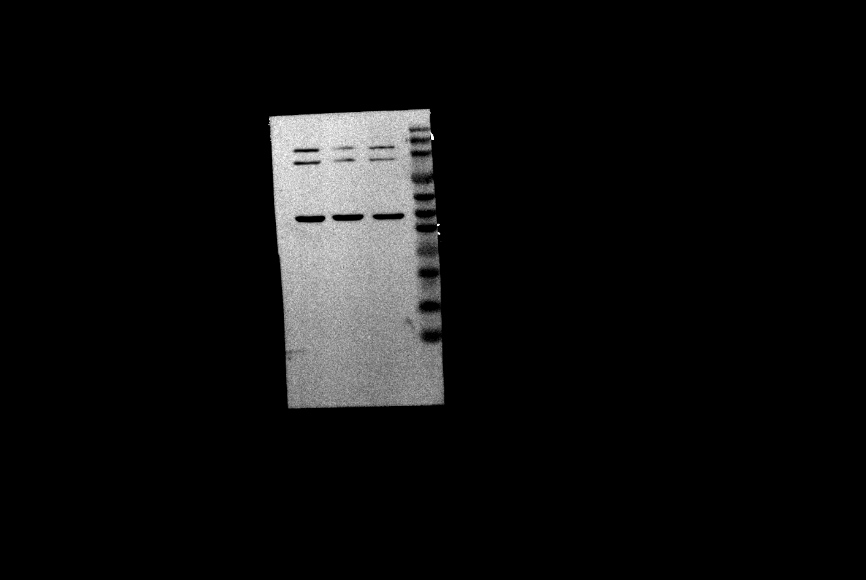
 **
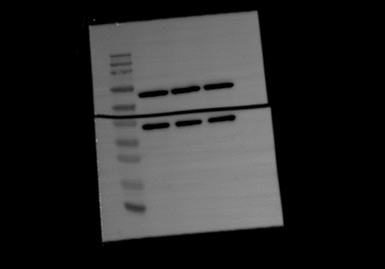
**

**
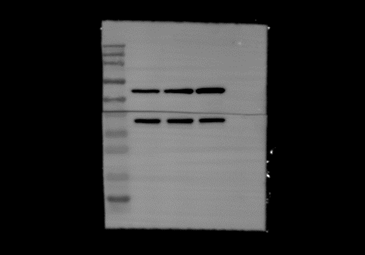
**

**Supplementary Figure 14. Original image of Figure 7G**

**Table 1**

| **Item** | **Company** | **Location** | **Product #** |
| --- | --- | --- | --- |
| Hexafluoroisopropanol | Macklin | Shanghai, China | H811026 |
| Fe_3_O_4_ | Macklin | Shanghai, China | I811859 |
| Polycaprolactone | Sigma-Aldrich | Taufkirchen, Germany | 440744 |
| Pyrrole | Macklin | Shanghai, China | P815709 |
| FeCl_3_ | Macklin | Shanghai, China | I811935 |
| p-Toluenesulfonyl chloride | Macklin | Shanghai, China | T821340 |
| RSC96 | Pricella | Wuhan, China | CL-0199 |
| Dulbecco's Modified Eagle Medium | Pricella | Wuhan, China | PM150210 |
| Fetal Bovine Serum | Gibco | Gaithersburg, United States | A5669701 |
| Penicillin-Streptomycin Solution | Pricella | Wuhan, China | PB180120 |
| Phosphate-Buffered Saline | Pricella | Wuhan, China | PB180327 |
| Trypsin EDTA (0.25%) | Gibco | Gaithersburg, United States | 25200072 |
| Polylysine | Sigma-Aldrich | Taufkirchen, Germany | P3513 |
| Hydrochloric acid | Aladdin | Shanghai, China | H399657 |
| PC12 | National Collection of Authenticated Cell Cultures | Shanghai, China | SCSP-517 |
| Recombinant Human Beta-NGF Protein | ABclonal | Wuhan, China | RP01792 |
| Paraformaldehyde | Biosharp | Hefei, China | BL539A |
| Triton X-100 | Beyotime | Shanghai, China | P0096 |
| Anti-Trkb antibody | Servicebio | Wuhan, China | GB11295-1 |
| Anti p-Trkb antibody | Abcam | Cambridge, UK | AB229908 |
| Anti AKT antibody | Servicebio | Wuhan, China | GB111114 |
| Anti p-AKT antibody | Servicebio | Wuhan, China | GB150002 |
| Anti BDNF antibody | Abcam | Cambridge, UK | AB205067 |
| Anti CREB antibody | Servicebio | Wuhan, China | GB111052 |
| Anti p-CREB antibody | Servicebio | Wuhan, China | GB114322 |
| Anti BDNF antibody | ABclonal | Wuhan, China | A4873 |
| Rat BDNF ELISA Kit | Multi Sciences | Hangzhou, China | EK3127 |
| Rat IFN-gamma ELISA Kit | Servicebio | Wuhan, China | GER0006 |
| Human/Mouse/Rat TGF-β1 ELISA Kit | Multi Sciences | Hangzhou, China | EK981-01 |
| Rat IL-2 ELISA Kit | Multi Sciences | Hangzhou, China | EK302/2-01 |
| Rat IL-4 ELISA Kit | Multi Sciences | Hangzhou, China | EK304/2-01 |
| Rat IL-10 Uncoated ELISA Kit | Thermo Fisher | Waltham, United States | 88-50629 |
| Rat IL-17A ELISA Kit | Multi Sciences | Hangzhou, China | EK317/3 |
| Enzyme-linked Immunosorbent Assay Kit  For Interleukin 18 (IL18) | Cloud-Clone Corp | Wuhan, China | SEA064Ra |
| Rat IL-1 beta Tissue Culture Uncoated ELISA | Thermo Fisher | Waltham, United States | 88-6010A |
| Rat IL-6 Uncoated ELISA Kit | Thermo Fisher | Waltham, United States | 88-50625 |
| Rat TNF alpha Uncoated ELISA | Thermo Fisher | Waltham, United States | 88-7340 |
| Rat CXCL1/CINC-1/KC(IL-8) ELISA kit | Neobioscience | Shenzhen, China | ERC008 |
| BSA | Sigma-Aldrich | Taufkirchen, Germany | V900933 |
| TWEEN-20 | Beyotime | Shanghai, China | ST828 |
| Glutaraldehyde | Macklin | Shanghai, China | G849973 |
| Muscle fixative | Servicebio | Wuhan, China | G1111 |
| Goat anti-Rabbit IgG (H+L) Cross-Adsorbed Secondary Antibody, Alexa Fluor™ 488 | Thermo Fisher | Waltham, United States | A-11008 |
| Goat anti-Mouse IgG (H+L) Highly Cross-Adsorbed Secondary Antibody, Alexa Fluor™ 546 | Thermo Fisher | Waltham, United States | A-11030 |
| Donkey anti-Rabbit IgG (H+L) Highly Cross-Adsorbed Secondary Antibody, Alexa Fluor™ 546 | Thermo Fisher | Waltham, United States | A-10040 |
| Donkey anti-Mouse IgG (H+L) Highly Cross-Adsorbed Secondary Antibody, Alexa Fluor™ 488 | Thermo Fisher | Waltham, United States | A-21202 |
|  |  |  |  |

**Table 2**

| **Genes** | **Forward primer (5′ 3′)** | **Reverse primer (5′ 3′)** |
| --- | --- | --- |
| IL-1β | GTGATGAAAGACGGCACACC | TCCTGGGGAAGGCATTAGGA |
| IL-2 | CAGTGACGCTTGTCCTCCTT | AGCACCTGTAAGTCCAGCAA |
| IL-4 | CGTGATGTACCTCCGTGCTTGA | TCAGTGTTGTGAGCGTGGACTC |
| IL-6 | CTTCCAATGCTCTCCTAATG | GCCGAGTAGACCTCATAGTG |
| IL-8 | GAAGTTTTTGAAGAGGGCTGAGA | TTTGCTTGAAGTTTCACTGGCA |
| IL-10 | CCTGCTCTTACTGGCTGGAGTG | TGGGTCTGGCTGACTGGGAA |
| IL-17 | ACTTTCCGGGTGGAGAAGAT | CTTAGGGGCTAGCCTCAGGT |
| IL-18 | TGTGCACTCTCCTTACAACACA | GGGACAGCCAGTGTTCAGTCA |
| IFN-γ | ACCCACAGATCCAGCACAAAGC | CCAGAATCAGCACCGACTCCTT |
| TGF-β | CTGCTGACCCCCACTGATAC | AGCCCTGTATTCCGTCTCCT |
| TNF-α | ATGGGCTCCCTCTCATCAGT | GCTTGGTGGTTTGCTACGAC |
| GAPDH | CAGTGCCAGCCTCGTCTCAT | CAGCCTTGACTGTGCCGTTG |
